# Lentil-Derived Bioactives for Gastrointestinal Health: Potential Complementary Interactions Among Peptides, Resistant Starch, and Polyphenols

**DOI:** 10.3390/nu18091348

**Published:** 2026-04-24

**Authors:** Xingye Wei, Qianwen Sun, Chengxuan Li, Jinghan Wang, Muhammad Sajid Arshad, Hafiz A. R. Suleria

**Affiliations:** 1 School of Agriculture, Food and Ecosystem Sciences, Faculty of Science, The University of Melbourne, Parkville, VIC 3010, Australiasajid.arshad@unimelb.edu.au (M.S.A.); 2 School of Health Economics and Management, Nanjing University of Chinese Medicine, Nanjing 210023, China

**Keywords:** lentils, bioactive peptides, resistant starch, polyphenols, gut microbiota, intestinal barrier

## Abstract

Lentils (*Lens culinaris*; family: Fabaceae) are increasingly recognized as functional legumes with potential benefits for gut health because they provide bioactive peptides, resistant starch, and polyphenol-rich fractions within a shared food matrix. However, most existing studies have focused on individual lentil-derived compounds, and their matrix-dependent complementary interactions during digestion and fermentation remain insufficiently resolved. This review synthesizes current evidence on lentil-derived peptides, resistant starch, and polyphenols, with particular emphasis on their matrix-dependent complementary relationships, digestion-dependent transformation, microbial co-metabolism, and implications for intestinal barrier function. During gastrointestinal digestion and colonic fermentation, lentil proteins, resistant starch, and phenolic compounds undergo sequential transformation, yielding bioactive peptides, fermentable substrates, short-chain fatty acids (SCFAs), and phenolic metabolites that may collectively influence microbial composition and metabolic activity. Emerging evidence suggests that these interconnected processes may support gut health through microbiota–host crosstalk by modulating tight junction-related markers, reducing intestinal permeability, and maintaining epithelial homeostasis. Mechanistically, these effects have been associated with SCFA-mediated G protein-coupled receptor (GPCR) signaling, suppression of TLR4–NF-κB/MAPK inflammatory cascades, and activation of Keap1–Nrf2 antioxidant defenses, thereby attenuating oxidative stress and pro-inflammatory responses. Current evidence is more consistent with matrix-dependent complementary or convergent actions than with demonstrated synergy. At present, phenolic-rich fractions provide clear pathway-level evidence, whereas fermentation-linked carbohydrate effects are more strongly supported by microbiota- and in vivo-associated outcomes, and protein- or peptide-related mechanisms remain comparatively underdefined. Nevertheless, the evidence base remains limited by the scarcity of integrated studies, well-controlled human intervention trials, and factorial experimental designs capable of distinguishing complementary, additive, and truly synergistic effects among lentil bioactives. This review therefore highlights the need to move from describing coexisting beneficial effects toward formally testing interaction effects within physiologically relevant lentil matrices.

## 1. Introduction

Lentil (*Lens culinaris*; family: Fabaceae) is a diploid (2n = 14) self-pollinating annual crop. It is one of the oldest cultivated crops in the world and has long been used as an important ingredient in various cuisines, particularly in India and the Mediterranean region [1]. Major lentil-exporting countries include Australia, Canada, the United States, Turkey, and the United Arab Emirates [2]. As legumes, lentils are rich in protein and dietary fiber and low in fat. Lentil proteins are mainly composed of globulins, albumins, glutelins, and prolamins [3]. Lentils contain a range of amino acids, including glutamic acid, aspartic acid, arginine, leucine, lysine, phenylalanine, valine, isoleucine, alanine, and proline. They also contain bioactive compounds such as polyphenols [4]. Owing to their nutritional value, lentil-based foods have been investigated for their potential health benefits, with some studies suggesting protective effects against chronic conditions such as hypertension, diabetes, hypercholesterolemia, cancer, and cardiovascular disease. Lentils have also been studied for their potential to promote healthy dietary patterns by reducing food intake, enhancing satiety, and thereby supporting body-weight management, partly because of their low glycemic index and high fiber content [5,6].

As a functional food ingredient, lentils have been incorporated into a broad range of commercial products. Primary and secondary processing steps include cleaning, grading, packaging, dehulling, and splitting [7]. Tertiary processing involves milling, fractionation, and thermal treatment for the production of consumer-ready foods. In addition to their complementary protein profile and low-glycemic-index carbohydrates, lentils provide substantial amounts of dietary fiber and polyphenols, particularly in the seed coat (hull). Polyphenols in lentils, mainly proanthocyanidins, flavan-3-ols, and phenolic acids, contribute importantly to the antioxidant and anti-inflammatory activities of lentil extracts. Proteins, dietary fiber, and polyphenols may interact within the food matrix to influence the stability, bioaccessibility, and release of bioactive components during intestinal digestion [8]. In the current market, lentils are used as ingredients in a variety of value-added foods, including snacks, plant-based meat alternatives, low-alcohol gluten-free beers, dairy alternatives, bakery products, and pasta. The incorporation of lentil ingredients into these foods may improve sensory and textural properties while also enhancing their nutritional and health-promoting potential [9,10,11,12,13]. Given their nutritional value and health-promoting potential, lentils are considered a promising ingredient for the development of functional foods.

### 1.1. Gut Health and Microbiome as Emerging Nutritional Targets

Gut health refers to the maintenance of a balanced gut microbiota and appropriate host immune responses, thereby preserving mucosal barrier integrity and limiting excessive inflammation [14]. Disruption of host–microbiota interactions resulting from intestinal dysbiosis is considered an important contributor to the development of a range of chronic diseases, including obesity, inflammatory bowel disease (IBD), colorectal cancer, and diabetes [15]. Intestinal dysbiosis is usually characterized by a decrease in the diversity of commensal and beneficial bacterial communities, together with the abnormal proliferation of resident or opportunistic pathogenic microorganisms. These changes in microbial community structure can disrupt host immune homeostasis, impair immune function, and maintain the intestine in a chronic low-grade pro-inflammatory state [16]. A well-balanced intestinal microbiota, together with a prebiotic-rich and low-calorie diet, is associated with a lower risk of obesity. Prebiotic-enriched diets can beneficially modulate the composition and metabolic activity of the gut microbiota, leading enhanced satiety, improved intestinal motility, increased production of short-chain fatty acids (SCFAs) and prevention of gastrointestinal disorders, such as diarrhea and constipation [17]. Additionally, these dietary patterns may suppress pathogenic bacterial colonization in the gut. Furthermore, the intake of prebiotic-rich foods has been reported to stimulate immune responses, enhance the absorption of essential minerals (especially Zn and Se) and lower the risk of colon cancer [18]. Beyond gastrointestinal health, prebiotics also contribute to the mitigation of obesity and metabolic syndrome by lowering circulating glucose concentrations, reducing bile salt reabsorption and serum cholesterol levels, and improving insulin sensitivity [19,20,21].

Lentils are a good source of various bioactive compounds, including fermentable fibers, bioactive peptides, and phytochemicals [22]. Lentils possess a well-balanced amino acid profile that complements cereal proteins, thereby enhancing the overall nutritional quality of the diet. Moreover, they serve as a rich source of prebiotic carbohydrates, including sugar alcohols (SAs), raffinose family oligosaccharides (RFOs), fructooligosaccharides (FOS), and resistant starch (RS) [23]. RS functions as a form of dietary fiber rather than an available carbohydrate in the intestine and may therefore promote gut health. RS remains largely undigested in the upper gastrointestinal tract and can reach the colon intact. In the colon, RS is fermented by the gut microbiota, producing metabolites that help maintain intestinal homeostasis. Numerous studies suggest that RS has considerable prebiotic potential, which can improve postprandial blood glucose and insulin responses, enhance satiety, reduce serum cholesterol and fat accumulation, and help in weight management. Therefore, resistant starch in lentils may represent a promising functional component for modulating the gut microbiota and may contribute to alleviating microbiota-related metabolic disturbances [24,25,26,27].

### 1.2. Potential Complementary Interactions Among Lentil Bioactives: Peptides, Resistant Starch, and Polyphenols

Lentils are a good source of proteins, resistant starch, and polyphenols, and are increasingly recognized as bioactive-rich legumes with potential relevance to gut health and related physiological benefits [5,28]. Peptides, resistant starch, and polyphenols represent three major classes of lentil bioactives that may act in complementary ways within the lentil food matrix. At present, evidence in lentils is available mainly at the matrix and binary-interaction levels rather than for a fully resolved ternary system. In lentils, a substantial proportion of phenolics occurs in insoluble-bound form and is associated with the hull and cell-wall matrix, indicating that polyphenols are structurally embedded rather than existing as fully independent constituents within the seed [29]. Consistent with this matrix-dependent organization, studies on whole cooked lentil seeds and isolated cotyledon cells have shown that the intact cotyledon architecture simultaneously constrains starch and protein digestion, supporting the view that these macronutrient fractions are digested in an interdependent rather than isolated manner [30]. At the binary level, lentil protein isolate has been shown to interact with added model phenolics, including quercetin, rutin, ellagic acid, and cyanidin-3-O-glucoside, leading to changes in protein secondary structure and related functional or antioxidant properties [31,32]. These lentil-based findings suggest that potential complementary interactions among lentil bioactives are mechanistically plausible within the lentil matrix, although their direct three-way interplay during digestion and fermentation has been less frequently examined.

On this basis, this review examines the potential complementary interactions among lentil-derived peptides, resistant starch, and polyphenols, with emphasis on their structural characteristics, digestive behavior, metabolic transformation, and relevance to intestinal barrier function [31,32].

## 2. Methodology

This review was conducted as a narrative review with elements of systematic search to ensure comprehensive literature coverage. Peer-reviewed studies published mainly between 2010 and 2026 were considered, although a limited number of earlier seminal studies were included where necessary to support key gut-related signaling pathways and mechanistic interpretation. A structured search was performed in Web of Science, Scopus, PubMed, and ScienceDirect, with supplementary screening in Google Scholar. Reference lists of relevant articles and recent reviews were also screened for additional studies.

The search strategy used combinations of keywords and Boolean operators, including: (“lentil” OR “*Lens culinaris*”) AND (“peptides” OR “resistant starch” OR “polyphenols” OR “phenolic compounds”) AND (“gut health” OR “intestinal barrier”). Additional targeted terms such as “digestion”, “colonic fermentation”, “gut microbiota”, “short-chain fatty acids”, “tight junction”, “GPCR signaling”, “MAPK”, “Nrf2”, and “NF-κB” were used where relevant.

Inclusion criteria: studies were included if they investigated lentils (*Lens culinaris*) or lentil-derived fractions/products, particularly peptides, resistant starch, polyphenols, or their interactions, and/or reported outcomes related to digestion, fermentation, gut microbiota, SCFA production, intestinal barrier integrity, inflammation, or oxidative stress. Human, animal, *in vitro*, and *ex vivo* studies were eligible when relevant to the scope of this review.

Exclusion criteria: non-peer-reviewed publications, duplicate records, non-English papers, articles without accessible full text, and studies lacking direct relevance to lentil bioactives and gut health-related outcomes were excluded. For interpretive clarity, the reviewed evidence was further distinguished as lentil-specific evidence, broader legume-based evidence, general food matrix/mechanistic evidence, or the authors’ conceptual interpretation.

**Figure 1:**
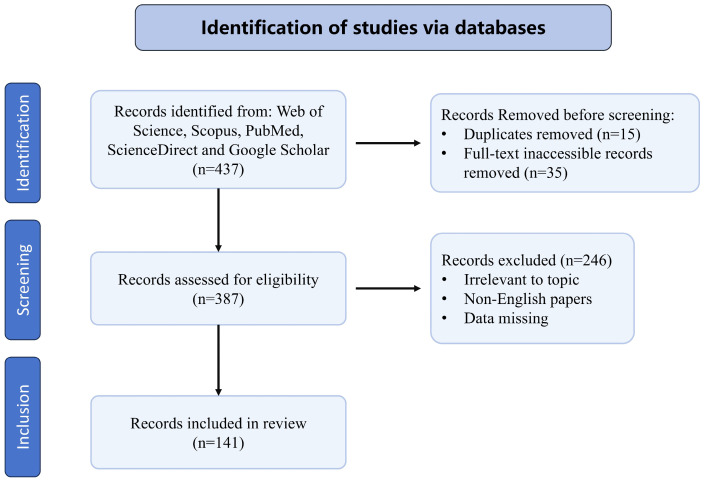
Flow diagram of study selection.

The database search initially retrieved 437 records from Web of Science, Scopus, PubMed, ScienceDirect, and Google Scholar. After the removal of duplicates and inaccessible records, a total of 387 records remained for screening. Following title and abstract screening, 246 records were excluded (irrelevant to topic, non-English papers, or data missing). Finally, 141 studies met the eligibility criteria and were included in the review. The flow diagram for study selection is shown in Figure 1.

## 3. Lentil Composition and Processing Influencing Bioactive Formation

### 3.1. Nutritional Composition and Bioactive Profile of Lentils 

Lentils are nutrient-dense pulses whose functional relevance to gut health arises not only from their overall nutritional value, but also from specific seed fractions that serve as sources of peptide precursors, resistant starch, and polyphenolic compounds. Lentils contain a protein range of around 25–30%, 63% carbohydrate, and only about 1% fat. They are also rich in dietary fiber, with reported levels ranging from 11% to 31% [7]. The protein content in the cotyledons is approximately twofold higher than that in the seed coat of lentils [33]. Additionally, lentils contain low-molecular-weight carbohydrates (LMWCs), which include raffinose-family oligosaccharides, sugar alcohols, fructooligosaccharides, and various mono- and disaccharides. These compounds are metabolized by intestinal microbiota in the large intestine and may exert beneficial effects on human health [34]. Among major pulse crops, lentils have been reported to contain higher dietary fiber levels than kidney beans, pigeon peas, chickpeas, and black gram [35]. Lentil proteins also exhibit a relatively balanced amino acid composition, with appreciable levels of essential amino acids such as lysine (1.80 g/100 g), leucine (1.87 g/100 g), and isoleucine (1.12 g/100 g), supporting their nutritional value as a plant protein source. However, sulfur-containing amino acids such as methionine (0.22 g/100 g) and cysteine (0.34 g/100 g) are present at relatively low levels. This pattern is consistent with that of other legume crops and suggests that lentils can complement cereal proteins in mixed diets, thereby improving overall protein quality [36]. While these general compositional features establish the nutritional value of lentils, the fractions most directly relevant to gut health are those associated with peptide generation, resistant starch delivery, and processing- and digestion-related changes in polyphenols.

Table 1 summarizes the major lentil constituents associated with peptide precursors, resistant starch, and polyphenol-rich seed fractions, together with their tissue distribution and representative processing-associated changes. Lentil proteins are presented in terms of their principal storage fractions, particularly globulins and albumins, because these fractions constitute the primary substrates for the generation of bioactive peptides during enzymatic hydrolysis and gastrointestinal digestion. Starch-related characteristics are described in terms of total starch, amylose, and resistant starch, with emphasis on the fact that digestive behavior is determined not only by chemical composition but also by the structural integrity of the cotyledon cell matrix. The table also highlights the preferential localization of polyphenols in the hull/seed coat fraction, including phenolic acids, flavan-3-ols, anthocyanidins, and condensed tannins, and indicates how dehulling and other processing treatments influence their abundance, retention, and bioaccessibility. Taken together, these quantitative and structural features provide a compositional basis for understanding how lentil-derived peptides, resistant starch, and polyphenols may be differentially released, transformed, and metabolized during digestion and colonic fermentation, thereby shaping their potential contributions to gut health.

For gut health, the functional value of lentil bioactives is better predicted by colonic availability and microbial transformability than by compositional abundance alone. Lentils are chemically rich in several classes of bioactives, including polyphenols, resistant starch, and other non-digestible carbohydrates, as well as encrypted bioactive peptides released during digestion, processing, or fermentation. From a gut health perspective, their relevance should not be judged solely by abundance, but by colonic delivery, microbial accessibility, biotransformation, and capacity to influence epithelial barrier and mucosal immune signaling. In this respect, polyphenols and non-digestible carbohydrate fractions, including resistant starch, appear to be the lentil bioactives most directly linked to colonic delivery and microbiota-mediated functionality, whereas evidence for the gut health relevance of lentil-derived peptides remains comparatively limited, although recent studies suggest emerging potential. Lentil compositional studies consistently show substantial phenotypic variation across cultivar, seed coat color, growing environment, and analytical method, which partly explains why absolute values reported for phenolics or other phytochemicals differ markedly between studies. Moreover, processing exerts a strong modifying effect: dehulling can remove a large proportion of hull-localized phenolics, while cooking, soaking, germination, and fermentation may either reduce extractable compounds or increase bioaccessibility by releasing bound forms [5,26,43,44].

Among lentil bioactives, polyphenols are the most consistently characterized at the compositional level. However, their relevance to gut health depends less on total abundance than on site-specific distribution and metabolic fate. Lentil seed coats contain abundant flavonoids, catechins, procyanidins, and tannin-type compounds, whereas cotyledons contain relatively higher levels of non-flavonoid phenolic acids. As a result, dehulling can substantially reduce the pool of phenolics available for downstream gastrointestinal transformation. This distinction is important because poorly absorbed parent polyphenols and bound phenolics can reach the colon, where they are further metabolized by the gut microbiota into smaller bioactive phenolic derivatives that may attenuate oxidative stress, inhibit NF-κB-driven inflammation, and support barrier function. Importantly, studies that compare total phenolic content across genotypes do not necessarily predict gut health functionality, because the apparent ranking of “high-phenolic” lentils can vary depending on extraction solvent, whether free or bound fractions are measured, and whether the sample is analyzed as whole seed or hull fraction. Thus, for evaluating gut health relevance, the hull-associated phenolic fractions released during digestion are likely more informative than bulk total phenolic content alone [5,45,46,47].

At the general mechanistic level, resistant starch is relevant to gut health because it escapes small-intestinal digestion and serves as a fermentable substrate for colonic microbiota. Its fermentation is associated with short-chain fatty acid production, particularly acetate and butyrate, which have been linked to epithelial barrier regulation and the suppression of intestinal inflammation [48]. In lentils specifically, direct evidence comes from both whole food and resistant-starch-focused studies. In mice, cooked red lentils increased microbial diversity, enriched SCFA-producing genera such as *Prevotella, Roseburia,* and *Dorea*, elevated fecal SCFAs, and improved barrier-related outcomes, although these findings were obtained from whole lentils rather than isolated resistant starch [49,50]. More direct lentil-specific evidence shows that resistant starch derived from lentils undergoes structure-dependent fermentation, with differences in multiscale architecture altering microbial succession and fermentation output in vitro [27]. In addition, pulse-derived resistant starch studies that included a lentil fraction further support beneficial effects on gut microbiota and intestinal health, but these should be interpreted as pulse-level rather than lentil-only evidence [26]. Taken together, current evidence suggests that resistant starch is likely to be an important contributor to lentil-associated microbiota modulation and barrier-related outcomes, while not excluding contributions from other lentil constituents.

The evidence for lentil-derived peptides should be discussed more cautiously. Their bioactivity is increasingly supported in hydrolysate studies, especially with respect to antioxidant, ACE-inhibitory, and enzyme-inhibitory effects, and fermentation may also improve protein digestibility and peptide release. However, compared with resistant starch and polyphenols, the evidence linking lentil peptides specifically to gut barrier protection or microbiota remodeling remains relatively preliminary. Much of the current literature is based on *in vitro* hydrolysis systems or functional characterizations outside the intestinal context, and the actual peptides that survive digestion and reach intestinal targets *in vivo* are still insufficiently defined. That said, the peptide fraction should not be dismissed: fermentation-based studies suggest that lentil proteins can be transformed into more bioaccessible peptide pools, and recent work using dynamic gut models indicates that fermented red lentil proteins isolates can influence the gut microbiota. At present, however, peptides are best framed as an emerging complementary bioactive class, potentially acting in concert with resistant starch and polyphenols, rather than as the principal gut-active component of lentils [51,52,53,54].

Overall, the key comparative point is that the most abundant lentil bioactives are not automatically the most relevant for gut health. For this specific physiological endpoint, the current evidence suggests a functional structure in which resistant starch provides the dominant microbiota-fermentable substrate, polyphenols provide redox- and inflammation-modulating co-effectors whose activity depends strongly on release and microbial biotransformation, and peptides represent a promising but still under-validated third layer of activity. This also helps explain why whole lentils often perform better than purified fractions alone: gut benefits are likely generated by the interaction of fermentable carbohydrates with phenolic and peptide components, rather than by any single compound class in isolation [26,27,47,49,51].

### 3.2. Processing-Induced Modifications in Peptides, Resistant Starch, and Polyphenols 

Processing can substantially modify the peptide, resistant starch, and polyphenol fractions of lentils. Current evidence indicates that lentil processing includes a range of physical, chemical, enzymatic, and biological approaches that alter structural organization and functional properties through distinct mechanisms, thereby affecting the nutritional and technological characteristics of processed lentil components.

Existing approaches for modifying lentil proteins and peptide precursors include traditional heat treatment, high-pressure homogenization, enzymatic hydrolysis, microbial fermentation, and related methods. Among these, heat treatment is one of the most widely studied methods for modifying lentil proteins, which serve as precursors of bioactive peptides. Heat treatment can induce time- and temperature-dependent changes in protein structure. For example, red lentil protein isolate (RLPI) subjected to prolonged heating (0–24 h) at 85 °C showed a progressive decrease in molecular weight and ultimately formed fibrillar aggregates at pH 2 and particulate aggregates at pH 7. These differences in aggregation morphology are mainly attributed to changes in protein charge state under different pH conditions, which in turn affect intermolecular interactions. With increasing temperature and treatment time, lentil proteins undergo partial conformational unfolding, disrupting secondary and tertiary structures originally stabilized by hydrogen bonds, hydrophobic interactions, and disulfide bonds, and exposing additional hydrophobic groups and thiol sites. This structural loosening promotes molecular recombination and the formation of thermally induced aggregates, ranging from soluble assemblies to insoluble macromolecular complexes. Such aggregation behavior not only alters protein molecular structure but also improves functional properties, including gel strength, network integrity, and water-holding capacity. The effects of heat treatment on lentil proteins are also highly time-dependent, as longer heating further promotes aggregate formation and produces denser and more stable three-dimensional gel networks [55]. Hall et al. (2021) [56] systematically examined the structural and functional changes in lentil proteins under heat treatment and high-pressure processing (HPP). The results showed that both treatments significantly modified protein molecular structure and gel properties. Heat treatment and HPP disrupted the original secondary and tertiary structures of lentil proteins, promoted denaturation, and enhanced aggregation, cross-linking, and molecular rearrangement under high-concentration conditions, thereby forming gels with greater strength and structural stability. In particular, the storage modulus (G′) of heat-treated samples generally increased by 1–2 orders of magnitude relative to untreated samples, indicating that the lentil protein gel network became more compact and exhibited greater elasticity and structural support capacity. High-pressure treatment showed a similar strengthening effect [56].

Although heat treatment and pressure-based processing are often used to modify lentil proteins for improved gelation or textural functionality, direct lentil evidence indicates that these treatments may also compromise solubility and selected interfacial properties under certain conditions. In the same comparative study that included lentil proteins, both heat treatment and HPP lowered solubility at low protein concentration, while changes in emulsifying and foaming properties varied with treatment type and protein source [56]. Similarly, in yellow lentil protein concentrate, HPP effects depended on pressure, holding time, and process pH: lower-pressure treatments at neutral pH improved solubility, whereas increasing pressure increased surface hydrophobicity and altered emulsion characteristics [57]. Thermal processing of lentil proteins have also been shown to modify secondary structure, increase hydrophobic surface exposure, and expose sulfhydryl groups, indicating that excessive or poorly controlled treatment may shift lentil proteins away from the structural state most favorable for interfacial functionality [58]. Therefore, when discussing processing-induced modification of lentil proteins, it is more appropriate to frame heat and pressure treatments as condition-sensitive tools that may either improve or impair functionality depending on the processing window and the target application.

Processing-induced modification in lentils is not confined to proteins, as the starch fraction is likewise highly process-responsive. Evidence from isolated resistant starch fractions shows that different treatments can substantially restructure lentil starch. In Laird lentils, resistant starch isolated after germination, fermentation, microwaving, conventional cooking, or autoclaving displayed clear differences in molecular weight, hydrodynamic radius, crystallinity, and double-helical order, indicating that processing alters starch structure well beyond simple quantitative retention or loss. Evidence from high-pressure treatment likewise confirms this sensitivity: in lentil starch dispersions, treatment at 600 MPa for 10 min completely gelatinized the starch, increased water-holding capacity and gel strength, and raised the resistant starch fraction from 5.0% to 6.8%. However, results from whole-seed and food-matrix systems show that the nutritional consequence of processing cannot be inferred from isolated starch behavior alone. In whole cooked lentils, shorter cooking preserved more intact cell clusters and slowed amylolysis, whereas longer cooking promoted tissue disintegration and accelerated starch digestion. Similarly, in red lentil pasta, heat treatment of grains or flour and extrusion-cooking increased starch susceptibility to α-amylase and reduced the slowly digestible starch fraction. Taken together, these findings suggest that processing does not uniformly increase or decrease resistant starch in lentils; rather, it redistributes starch among structurally protected, slowly digestible, and more enzyme-accessible states according to treatment severity and the extent of matrix disruption [30,59,60,61].

Lentil polyphenols are also highly sensitive to processing, although its predominant effect is better understood as compositional redistribution and transformation rather than a simple rise or decline in total phenolics alone. In Pardina lentils, soaking, cooking, and industrial dehydration altered a phenolic profile comprising 35 identified compounds, indicating that both domestic and industrial treatments can alter the qualitative phenolic composition of lentils. This interpretation is further supported by fraction-based analyses showing that cooking and germination modify the distribution of phenolic compounds across lentil dietary fiber fractions, with phenolics remaining largely associated with the insoluble fiber fraction after processing. At the same time, the direction and magnitude of these changes are clearly method-dependent. In lentils, pressure and microwave cooking increased total phenolic content in methanolic extracts, whereas pot boiling and slow cooking reduced it; however, the same study also found decreases in both free and bound phenolic acids, indicating that extractable total phenolics and individual phenolic subclasses do not necessarily respond in parallel. Earlier work likewise showed that germination and fermentation altered low-molecular-weight phenolic compounds in lentils, with fermentation increasing some phenolic constituents and generating metabolites not detected in raw seeds, while germination produced marked structural changes in procyanidin-type compounds [62,63,64,65].

A further processing-related shift arises from seed fractionation: studies on lentil seed fractions generally show higher phenolic content and antioxidant activity in hulls than in whole or dehulled samples, indicating that dehulling substantially changes the phenolic profile and antioxidant potential of the remaining cotyledon-rich material. Overall, the effect of processing on lentil polyphenols is best described as matrix-dependent redistribution, liberation, and transformation, which may in turn influence the forms in which these compounds are delivered to the colon and become available for subsequent microbial metabolism [47,63,66].

### 3.3. Impact of Digestion and Colonic Fermentation on Bioactive Release 

Gastrointestinal digestion and colonic fermentation are central biological stages governing the release, accessibility, and subsequent transformation of lentil-derived bioactives. Rather than serving as passive terminal events, these processes shape the intestinal availability of peptide fragments, starch-derived substrates, and phenolic compounds, as well as their potential contributions to gut-related functionality.

During upper gastrointestinal digestion, lentil proteins are progressively hydrolyzed into smaller peptides and amino acid-rich fragments, and the extent of this release is strongly influenced by matrix form and digestive conditions. In green lentils, simulated digestion showed higher protein hydrolysis in lentil protein isolate than in lentil flour or cooked lentils, and LC–QTOF/MS further indicated that both cooking history and gastrointestinal conditions altered the peptide profile generated during digestion [67]. A smaller body of work has also shown that selected lentil-derived peptides can remain functionally stable after simulated gastrointestinal exposure, including peptides with antioxidant and ACE-inhibitory potential [68]. These observations indicate that digestion acts not only as a degradative process, but also as a peptide-releasing and peptide-selecting stage in lentils.

For starch fractions, the key consequence of digestion is the partitioning between starch that is hydrolyzed in the small intestine and starch that escapes digestion and reaches the colon. In whole cooked lentils, shorter cooking times preserve more intact cotyledon cell clusters and are associated with slower *in vitro* amylolysis and proteolysis, showing that the physical integrity of the lentil matrix helps determine how much starch remains microbiota-accessible [30]. Once delivered to the colon, lentil-derived resistant starch undergoes structure-dependent fermentation. In particular, resistant starches derived from untreated and autoclaved lentil starch show different multiscale structural changes during fermentation and are associated with different microbial succession patterns and SCFA outputs [27]. More broadly, resistant starch fermentation is widely associated with SCFA generation and microbiota-dependent gut effects. In lentils, however, the strongest current evidence relates to fermentation behavior and metabolite output, whereas downstream barrier- or inflammation-related outcomes are still more often inferred from broader resistant-starch literature than from lentil-specific intervention studies [27,48,69].

Polyphenols show a particularly clear digestion–fermentation sequence, although the available lentil studies are still concentrated on hull fractions. In lentil hull systems, simulated digestion progressively releases polyphenols and generates digestive products that retain antioxidant activity, show evidence of epithelial transport in Caco-2 models, and remain sufficiently bioavailable to justify subsequent *in vivo* metabolism studies [70]. Complementary work on free and bound phenolics from Laird lentil hulls further showed that digestion-derived phenolic mixtures retained anti-inflammatory activity through NF-κB- and Keap1–Nrf2-related signaling [47]. In a co-culture model, digestive products from lentil hull extracts were also associated with intestinal barrier maintenance and reduced inflammatory responses [71]. Beyond lentils, broader work on gut bacterial polyphenol metabolism suggests that non-absorbed dietary polyphenols may undergo further deglycosylation, dehydroxylation, and ring-cleavage reactions in the colon, thereby generating lower-molecular-weight metabolites with altered bioavailability and biological relevance. In this sense, digestion appears to initiate phenolic release in lentils, whereas colonic fermentation is likely to extend and diversify the pool of phenolic derivatives that ultimately interact with the host [72].

Taken together, current findings support a stage-dependent view of lentil bioactive release. Upper gastrointestinal digestion primarily governs the initial liberation and accessibility of peptides, starch fractions, and phenolics, whereas colonic fermentation further shapes the metabolite profile, particularly for resistant starch and non-absorbed phenolic compounds. Overall, the available evidence supports an evidence-guided framework for interpreting lentil bioactive release and transformation, rather than a fully resolved lentil-specific interaction model.

As summarized in Table 2, the gut-related effects of lentil-derived bioactives depend strongly on processing history, digestion, colonic fermentation, and the food matrix. These factors influence peptide release, resistant starch accessibility, and the transformation or bioaccessibility of polyphenols, although the strength of evidence varies considerably across experimental models. Notably, dynamic systems such as SHIME^®^ (Simulator of the Human Intestinal Microbial Ecosystem) are useful for capturing microbiota-mediated responses to digestion-derived lentil components in a more integrated manner. These findings suggest that the intestinal effects of lentil bioactives arise from context-dependent transformation rather than from fixed intrinsic properties of the raw ingredients.

## 4. Lentil Bioactive Peptides and Their Role in Gut Health 

### 4.1. Protein Composition and Peptide Generation Pathways

In lentils, the major protein fraction consists of storage proteins that act as key sources of nitrogen, carbon, and sulfur required during germination and subsequent seedling development. They are primarily localized in the cotyledons, where they are organized into small spherical protein bodies (2–4 μm) and a surrounding protein matrix that encapsulates starch granules [37,86]. Lentil proteins are generally classified into four major fractions: globulins (70%; 7S and 11S, saline-soluble), albumins (16%; water-soluble), glutelins (11%), and prolamins (3%) [87]. Multiple polypeptide chains link together to form these proteins, where glutelins have 4 chains, albumins have 13 chains and prolamins have 10 chains. The molecular weights of these protein fractions are relatively low, in the range of 17–46 kDa for glutelins, around 20 kDa for albumins, and 16–64 kDa for prolamins. Globulins can be further classified into two heterogeneous oligomeric proteins, namely legumin and vicilin [88]. Legumin (11S) typically has a molecular weight in the range of 320–380 kDa and consists of six subunits, each comprising an acidic polypeptide (~40 kDa) and a basic polypeptide (~20 kDa) linked by disulfide bonds [3]. Vicilin (7S fraction) has a molecular weight in the range of 50–60 kDa and is composed of glycosylated subunits, which contain approximately 3% carbohydrates [89]. In the cotyledons, legumin and vicilin represent 21.4% and 15.3% of the total protein content, respectively. In the isolated globulin fraction, the ratio of legumin to vicilin was approximately 1.6:1 [90]. Compared with globulins, albumins (2S proteins) have lower molecular weights and more hydrophilic surfaces, which contribute to their higher water solubility [91]. Lentil albumins consist of 13 polypeptides with molecular weights below 30 kDa. The major components include albumin 2 (~26 kDa), albumin 1 (~15 kDa), and a disulfide-linked albumin polypeptide (~14 kDa), as well as metabolically important proteins such as lipoxygenases (95–97 kDa) and phytohemagglutinin (~32 kDa) [90]. Reported lentil protein profiles may vary among studies because differences in genotype and protein extraction methods can substantially influence the observed composition [37].

Recent studies on germination, targeted hydrolysis, simulated digestion, and colonic fermentation collectively suggest that lentil peptide generation follows a multistage pathway. Lentil storage proteins (mainly 7S vicilins and 11S legumins) dominate the globulin fraction and constitute the primary precursors of bioactive peptides. Their hierarchical structure and subunit organization are linked to their techno-functional and nutraceutical properties [90]. Together, these observations indicate that lentil storage proteins provide the structural basis for subsequent peptide release.

During germination, endogenous proteases are strongly upregulated and progressively cleave these storage proteins into lower-molecular-weight fractions. For example, Bautista-Expósito et al. (2021) demonstrated that 4–6 days of lentil germination followed by an in vitro INFOGEST 2.0 protocol digestion markedly increased the release of peptides that retained activity after gastrointestinal digestion, with enhanced ACE-inhibitory and antioxidant effects relative to ungerminated seeds [92,93]. Mirzapour-Kouhdasht et al. (2025) [94] quantified the structural transition after germination. After six days of germination, in vitro protein digestibility increased in ranges from around 16–17% to 25–26%, the peptidome shifted toward fractions below 3 kDa, and both ACE-inhibitory and antioxidant capacity improved after digestion. These findings indicate that germination can convert lentil proteins into more digestible, peptide-enriched substrates with enhanced bioactivity after digestion [94]. Thus, germination appears to act as an upstream priming step that enhances protein susceptibility to subsequent peptide generation.

Exogenous enzymes applied during downstream processing further shape the lentil peptidome. Limited hydrolysis of lentil flour with pancreatin (degree of hydrolysis: 4–8%) partially depolymerizes vicilin and legumin subunits, enriching 0.4–1 kDa peptides while leaving some basic 11S subunits intact. This structural modulation is accompanied by an increase in ABTS and DPPH radical scavenging, ferric-reducing power, and Fe^2+^-chelating capacity, with low-DH hydrolysates showing the highest antioxidant activities [95]. Rezvankhah et al. (2023) [51] used sequential alcalase and flavourzyme to generate lentil protein hydrolysates containing short peptides enriched in hydrophobic and aromatic residues. These peptides exhibit strong antioxidant, ACE-inhibitory, and α-glucosidase/α-amylase-inhibitory activities, together with good gastrointestinal stability, which illustrates that proper enzyme selection and reaction control can steer peptide length and composition toward defined bioactivities [51]. Martoccia et al. (2025) further showed that enzymatic hydrolysis, microbial fermentation, and processing can induce structural changes (partial unfolding, exposure of cleavage-susceptible regions and enrichment of specific sequence motifs), which are key determinants of both peptide release kinetics and bioactivity after digestion [96]. These findings suggest that targeted downstream processing does not merely increase peptide yield, but can also shape peptide size, compositional features, and associated functionality.

Gastrointestinal digestion and colonic fermentation represent the terminal biological stages in the peptide generation pathway. Mirzapour-Kouhdasht et al. (2025) reported that pepsin–pancreatin digestion of germinated and non-germinated lentil proteins further shifted the peptide profile toward oligomers and di-/tripeptides, with low-molecular-weight fractions concentrating ACE-inhibitory and antioxidant activity and exhibiting improved bioaccessibility [94]. Once undigested protein and peptides reach the colon, microbiota-mediated transformation further refines the peptide and metabolite profile. Beyond peptide release itself, the fraction that escapes upper-gut digestion may continue to exert biological effects in the colon. Boachie et al. (2023) [97] demonstrated that undigested glycated lentil proteins surviving upper-gut digestion substantially modulated gut microbial composition in the Simulator of the Human Intestinal Microbial Ecosystem (SHIME) and batch fermentations, even though short-chain fatty-acid profiles were relatively stable. This highlights how processing can shift the balance between absorbed peptides and those delivered to the microbiota [97]. Complementarily, Mastrolonardo et al. (2025) showed that bioactive peptides generated by lactic acid bacteria fermentation of red lentil protein isolate can influence microbial community structure and metabolite production when subjected to dynamic *in vitro* colonic fermentation, which further suggests that lentil peptides may act not only as systemic effectors but also as modulators of the gut ecosystem [52]. Accordingly, the potential gut-level relevance of lentil-derived peptides depends not only on their release during processing and digestion, but also on their persistence and transformation within the colonic environment.

### 4.2. Characterized Bioactive Peptides and Functional Properties

Recent research has provided increasing molecular-level evidence on the characterization of bioactive peptides derived from lentil proteins and the functional properties that emerge following controlled hydrolysis, processing, and digestion. Enzymatic hydrolysis remains the most well-characterized and well-controlled strategy for peptide generation. Pancreatin-generated hydrolysates of lentil flour have been shown to undergo a marked shift in molecular-weight distribution, with selective enrichment in <1 kDa peptide fractions corresponding to increased radical-scavenging and ferric-reducing activities. Detailed analyses using SDS-PAGE and size-exclusion chromatography confirmed that progressive hydrolysis disrupts the native 7S vicilin and 11S legumin assemblies, which may expose domains susceptible to cleavage, thereby enhancing solubility and surface reactivity [95].

Beyond biochemical characterization, recent integrative studies have linked peptide structural attributes to techno-functional properties critical for food applications. Vogelsang-O’Dwyer et al. (2022) [98] reported that pulse protein hydrolysates, including those from lentils, exhibited significantly improved solubility, emulsifying activity, and interfacial adsorption behavior. These properties are attributed to the generation of shorter peptide chains with modified hydrophobic–hydrophilic balance and lower steric hindrance. Such peptides demonstrated enhanced dispersibility and surface activity, enabling more effective interactions with lipid and air–water interfaces [98]. Shevkani et al. (2024) [90] highlighted that lentil proteins, due to their globulin-rich profile and susceptibility to processing-induced structural loosening, provide a particularly favorable substrate for generating bioactive peptides with both nutritional and technological value. Their analysis emphasized that controlled protein unfolding, whether achieved through pH shifting, thermal treatment, or enzymatic action, plays a decisive role in determining peptide release kinetics, sequence enrichment, and ultimate functional performance [90].

Lentil protein hydrolysates can improve not only antioxidant and antihypertensive activities but also foaming and emulsification characteristics important for plant-based food formulation. For example, Lopes et al. (2023) showed that processing technologies such as heating and microfluidization, when applied prior to enzymatic hydrolysis, significantly altered peptide profiles and improved functional properties in lentil protein hydrolysates, thereby linking structural characterization with actual application potential [99]. This evidence supports an experimentally substantiated model in which the structural modification of lentil globulins increases enzyme accessibility, enabling controlled enzymatic hydrolysis to release short-chain peptides enriched with sequence motifs that underlie their biological activity. These molecular attributes not only govern antioxidant and antihypertensive potential but also drive improvements in key techno-functional properties, including solubility and emulsification.

### 4.3. Mechanistic Actions on the Gut Epithelium, Inflammation, and Microbial Modulation

Lentils are increasingly recognized as a model pulse for probing how specific food matrices reshape the gut epithelium–immune–microbiota axis. Their hulls and cotyledons provide a dense source of fermentable dietary fiber, resistant starch, and phenolic compounds that escape digestion in the upper gut and become substrates for colonic microbes. A comprehensive review of lentil prebiotic carbohydrates outlined how α-galactooligosaccharides, resistant starch, and cell-wall polysaccharides from lentils selectively stimulate saccharolytic taxa and drive short-chain fatty acid (SCFA) production, thereby supplying energy to colonocytes and supporting epithelial barrier homeostasis [34]. This conceptual framework is increasingly supported by targeted *in vivo* studies. Using a dextran sulfate sodium (DSS) colitis model, Chen et al. (2025) [100] showed that soluble dietary fiber isolated from lentil hulls not only attenuates colonic inflammation and histological damage but also partially normalizes anxiety-like behavior through a gut–brain axis mechanism. The cellulase-modified lentil hull fiber produced a more porous, highly fermentable structure, shifted the microbiota away from potentially harmful taxa such as *Bacteroides* and *Escherichia*, increased beneficial genera including *Akkermansia* and *Allobaculum*, and was associated with lower colonic and systemic levels of LPS, IL-1β, IL-6, and TNF-α while enhancing IL-10 and brain-derived neurotrophic factor (BDNF) [100]. These findings support a framework in which structurally tailored lentil fibers strengthen epithelial barrier integrity and attenuate mucosal and neuroinflammation through microbiota-derived metabolites and modulation of host signaling, rather than acting merely as bulking agents.

Parallel lines of evidence indicate that lentil-derived phytochemicals can interact more directly with intestinal and immune cells. Panaro et al. (2024) examined extracts derived from lentil seed coats, a polyphenol-rich agro-industrial waste stream, and demonstrated that a microwave-assisted ethyl acetate extract downregulated TLR4 and iNOS expression, decreased IL-1 production, and upregulated IL-10 in LPS-activated Caco-2 cells, while exerting spasmolytic effects in *ex vivo* intestinal tissue [101]. This profile is consistent with a local anti-inflammatory, barrier-supporting action at the epithelial surface and suggests that lentil phenolics can modulate both innate immune receptors and downstream cytokine networks. The fiber fraction of lentils appears to converge on similar outcomes through a related but not identical mechanistic route. In rodent models, recent studies on green lentil hulls have shown improvements in gut health biomarkers together with reductions in markers associated with non-alcoholic fatty liver disease, partly mediated by increased SCFA production and favorable shifts in gut microbial composition. These observations further underscore the systemic reach of lentil-driven changes in the intestinal milieu [102].

Protein-derived components add a further layer of complexity. Boachie et al. (2023) [97] investigated undigested glycated lentil protein residues likely to reach the colon and found that, in batch fermentation systems, these residues substantially altered gut microbiota composition without markedly changing total SCFA or ammonia output. These findings suggest that lentil proteins processing may primarily influence the composition of microbial populations supported by lentil-derived nitrogen, rather than the overall magnitude of fermentation [97]. At the whole-diet level, human intervention studies are beginning to connect these mechanistic observations to clinically relevant phenotypes. A 12-week randomized trial on adults consuming 980 g/day of cooked green lentils showed improvements in fasting cholesterol and postprandial inflammatory responses, consistent with potential systemic anti-inflammatory effects of sustained lentil intake, even though the study did not profile the microbiome directly [103]. In a complementary design, Govindan et al. (2026) compared lentil proteins with whey and egg proteins during a 12-week resistance-training protocol and reported distinct shifts in gut microbiota composition and fecal SCFA profiles in the lentil protein group, indicating that lentil proteins, like their carbohydrate fractions, may act as modulators of microbial ecology and fermentation outputs in humans [104]. Taken together, these findings support a framework in which lentil fiber, resistant starch, phenolic extracts, and processed proteins may collectively strengthen epithelial barrier function, rebalance pro- and anti-inflammatory signaling, and reshape microbial communities toward SCFA-producing, potentially health-promoting profiles.

## 5. Resistant Starch and Polyphenols in Gut Microbiome Modulation

### 5.1. Structural Diversity and Fermentability of Lentil Starch

Lentil starch shows pronounced structural heterogeneity at molecular and granular scales, and this heterogeneity is a primary determinant of both digestibility in the small intestine and fermentability in the colon. Wang et al. (2014) showed that starches from different pea and lentil varieties differed in physicochemical characteristics closely related to digestive behavior, indicating that cultivar-dependent structural variation is already evident at the raw material level [105]. More directly, Zhao et al. (2024) [27] demonstrated that resistant starch prepared from untreated and autoclaved lentil starch underwent distinct multiscale structural transitions during dynamic *in vitro* fermentation, and that these differences were accompanied by divergent patterns of microbiota metabolism. These findings suggest that lentil-derived resistant starch should be viewed as a structurally conditioned fermentation substrate rather than as a chemically uniform residue [27].

This interpretation fits well within the broader pulse-starch literature. Pulse starches differ substantially in granule morphology, crystalline organization, molecular packing, and amylose proportion, and these features influence gelatinization, retrogradation, enzyme accessibility, and hydrolysis kinetics. As a result, processing and digestion do not merely reduce starch quantity; they also reshape the physical state of the material that remains available for microbial fermentation. In lentils, this means that cooking history, digestion-stage structural selection, and residual ordering are all likely to influence how efficiently microbial enzymes can access the substrate once it reaches the large intestine [106].

More generally, recent syntheses of resistant starch–microbiome interactions support the view that resistant starch functions as a processing-dependent ecological substrate. During upper gastrointestinal digestion, more accessible amorphous regions are preferentially hydrolyzed, whereas less accessible or more ordered domains become relatively enriched in the residue entering the colon. Subsequent fermentation then depends on the interaction between substrate structure, primary microbial degradation, and community-level cross-feeding. For lentils, the most relevant interpretation is therefore that starch fermentability emerges from the interplay between intrinsic starch architecture, processing-induced reorganization, and microbiota-mediated breakdown in the colon [48].

### 5.2. Microbial Fermentation of Resistant Starch and SCFA Production

Recent work has begun to clarify how lentil resistant starch behaves once it enters the colonic environment. Zhao et al. (2024) [27] reported that lentil resistant starch underwent progressive structural remodeling during fermentation, while microbial metabolic profiles shifted in parallel, indicating that lentil-derived resistant fractions are actively utilized rather than merely persisting as inert residues. This is mechanistically important because fermentation is not a single-step conversion; instead, the initial microbial attack on structurally resistant glucans generates smaller intermediates that can be further metabolized by other taxa, so substrate structure can shape both primary degradation and downstream cross-feeding [27].

Evidence from pulses more broadly strengthens the physiological relevance of this process. Kadyan et al. (2023) [26,107] showed in a humanized murine model of aging that resistant starches from dietary pulses modulated the gut metabolome in association with the microbiome, while related work from the same group linked pulse-derived resistant starch to improvements in intestinal health and reductions in inflammatory stress. These studies are not restricted to lentils alone, but they are highly informative because they indicate that legume-derived resistant starch can produce biologically meaningful microbiome–metabolome responses *in vivo* [26,107].

At the mechanistic level, once resistant starch reaches the colon, microbial fermentation becomes the principal biochemical process dictating its functional relevance. Resistant starch is not hydrolyzed in the small intestine and instead provides a fermentable substrate for colon-resident microbiota, which ferment resistant starch into a suite of metabolites dominated by short-chain fatty acids (SCFAs) including acetate, propionate, and butyrate. These metabolites represent a critical metabolic link between dietary structure and host physiology because they serve both as substrates for colonocytes and as signaling molecules that influence immune, metabolic, and barrier-related pathways. Fermentation of resistant starch by the resident microbiota is thus a necessary step in translating carbohydrate intake into host-relevant biochemical exposures [48,108].

The production of SCFAs from resistant starch is underpinned by a multi-stage catabolic cascade carried out by specialized bacterial taxa. Primary degraders in the gut break down complex and structurally resistant carbohydrates into smaller oligosaccharides, which are then further fermented by other community members into SCFAs. Broad taxonomic surveys indicate that RS fermentation supports saccharolytic and SCFA-associated bacteria such as *Bifidobacterium*, as well as butyrate-producing taxa within the Lachnospiraceae and Ruminococcaceae families [109]. In vitro and animal studies consistently show that supplementing diets with RS increases fecal concentrations of acetate, propionate, and especially butyrate. The latter is a primary energy source for colonocytes and a key regulator of epithelial homeostasis [108,110].

Functional evidence for RS fermentation outcomes comes not only from biochemical profiling but also from intervention-based studies. In a humanized murine model colonized with human microbiota, pulse-derived resistant starch supplementation was shown to reshape the gut metabolomic profile, including increased SCFA levels, concomitant with shifts in microbial metabolic pathways. These metabolic outputs were observed even when changes in community taxonomic composition were modest, highlighting that functional metabolic responses can be decoupled from large-scale taxonomic shifts [107,111]. Similarly, randomized intervention studies in humans supplemented with different RS types reported increases in SCFA concentrations in fecal samples, along with improvements in metabolic markers such as weight loss and insulin sensitivity, linking microbiome fermentation outputs with systemic host phenotypes [112].

SCFAs exert multiple downstream biological activities once produced. Butyrate is the preferred energy source for colonocytes and can influence gene expression by modulating histone acetylation states and activating free-fatty-acid receptors that participate in both local and systemic signaling pathways [110,113]. Propionate and acetate also interact with host G protein-coupled receptors, contributing to the regulation of immune responses, gluconeogenesis, and lipid metabolism [114]. Beyond metabolic support, SCFAs have established roles in modulating inflammatory pathways and promoting epithelial barrier integrity by enhancing mucosal tight junction protein expression and increasing mucin production, thus providing a plausible mechanistic route from RS fermentation to barrier-related phenotypes [110]. Notably, although the vast majority of RS fermentation research has been conducted in the context of mixed dietary fibers or pulse-inclusive systems rather than lentil RS in isolation, the fundamental biochemical pathways and microbial processes are conserved. The capacity of lentil-derived resistant starch to be fermented into SCFAs, together with the influence of these metabolites on host pathways, is broadly consistent with patterns observed across studies of dietary resistant starch. This supports the interpretation of resistant starch fermentation as a key mechanistic node linking diet–microbiome–host outcomes [48,115].

### 5.3. Polyphenol Diversity, Metabolism, and Impact on Microbial Balance

In lentils, the clearest evidence concerns the localization and digestive fate of hull-associated phenolics. Mustafa et al. (2022) [5] reported that lentil polyphenols comprise several major classes, including phenolic acids, flavan-3-ols, proanthocyanidins, anthocyanidins, and related compounds, whereas Manco et al. (2023) [41] further showed that these phenolic pools are concentrated predominantly in the seed coat rather than the cotyledon. This tissue distribution is mechanistically important because phenolics retained in the hull are more likely to remain associated with cell-wall-rich structures during digestion and therefore to enter the distal gut as bound or poorly extractable forms, rather than being fully released and absorbed in the upper gastrointestinal tract [5,41].

Several lentil-focused studies further indicate that digestion does not abolish phenolic activity, but instead reshapes it into new bioactive mixtures. Guo et al. (2022) [47] systematically characterized free and bound phenolics from Laird lentil hulls and evaluated their simulated digestion products using an intestinal epithelial inflammation model. The results showed that digestion-derived phenolic fractions retained significant anti-inflammatory activity and modulated NF-κB and Keap1–Nrf2 signaling in HT-29 cells, indicating that bioactivity can persist despite extensive chemical modification during digestion [47]. Complementary findings were reported by Peng et al. (2022), who demonstrated that lentil hull extracts and their digestive products attenuated inflammatory responses in a Caco-2 and RAW264.7 co-culture system through MAPK and NF-κB pathway modulation, supporting the view that digestion generates functionally active phenolic pools rather than inert degradation products [71]. Extending this line of evidence, Guo et al. (2023) showed that lentil hull polyphenols are bioaccessible, selected digestion-released compounds can cross Caco-2 monolayers, and *in vivo* metabolism in rats involved extensive phase I/II biotransformations, including hydroxylation, methylation, glucuronidation, and sulfation [70]. These studies indicate that lentil phenolics remain biologically relevant after digestion, although in chemically transformed rather than native forms.

Across pulses and other plant matrices, this pattern is consistent with the broader behavior of structurally associated phenolics. Seed coats and other cell-wall-rich tissues frequently contain substantial insoluble-bound or non-extractable phenolic fractions, meaning that solvent-extractable phenolics alone may underestimate the pool that is ultimately relevant to lower-gut exposure. In this context, the location of phenolics within the food matrix, their degree of binding, and their release during digestion may be more informative than bulk phenolic content alone when discussing gut-relevant activity. For lentils, this broader framework helps explain why hull-associated phenolics are especially important when considering interactions with the colonic microbiota [116].

More broadly, microbial metabolism is a central determinant of the ultimate biological fate of dietary polyphenols. General mechanistic reviews show that many polyphenols undergo only limited host-driven transformation in the upper gastrointestinal tract, whereas the colonic microbiota can mediate extensive scaffold-dependent reactions that generate smaller metabolites with altered bioavailability, signaling capacity, and ecological effects. These metabolites may in turn influence inflammatory tone, host–microbe signaling, and the relative abundance or activity of specific microbial groups [72]. In addition to this chemical transformation, polyphenols and their microbial metabolites can exert selective pressures on the gut microbiome, shaping microbial community balance through ecological filtering rather than broad antimicrobial effects. Integrative reviews of polyphenol–microbiota interactions further suggest that phenolic substrates can favor taxa capable of phenolic catabolism while constraining opportunistic or pro-inflammatory microorganisms, thereby contributing to functional shifts in microbial metabolism and inflammatory tone. In this context, lentil polyphenols are best interpreted as regulatory substrates whose primary contribution lies in modulating microbial function and host–microbe signaling rather than serving as major energy sources [117]. Taken together, current evidence suggests a structure in which lentil polyphenols undergo limited but functionally meaningful modification during digestion, followed by extensive microbiota-mediated transformation in the colon. The resulting phenolic metabolites shape microbial balance and inflammatory signaling, positioning colonic fermentation as the critical processing stage that defines the impact of polyphenols on gut microbial ecology.

### 5.4. Combined Effects of RS and Phenolics on Gut Barrier Integrity

Gut barrier integrity is best viewed as a system-level outcome shaped by the interaction of microbial metabolism, epithelial signaling, and inflammatory regulation. In lentils, resistant starch and phenolic fractions appear to contribute to this process through distinct but biologically compatible routes within the food matrix. Current lentil evidence does not yet demonstrate a direct interaction effect between isolated resistant starch and phenolics in a factorial sense; however, it does support the presence of two barrier-relevant pathways that converge at the intestinal interface.

With respect to lentil-derived carbohydrates, Zhao et al. (2024) [27] showed that lentil resistant starch undergoes progressive structural remodeling during dynamic *in vitro* fermentation, accompanied by shifts in microbiota metabolism, indicating that lentil resistant starch functions as a structurally contingent fermentation substrate rather than a passive digestion residue. Although this study did not directly assess barrier endpoints, it provides an important mechanistic basis for interpreting lentil resistant starch as a contributor to the colonic metabolic environment from which barrier-relevant signals may arise [27]. On the phenolic side, Guo et al. (2022) demonstrated that digestive products derived from free and bound phenolics of lentil hulls retained anti-inflammatory activity in HT-29 cells and were associated with the coordinated modulation of NF-κB and Keap1–Nrf2 signaling [47]. Peng et al. (2022), using Caco-2 monolayers and Caco-2/RAW264.7 co-culture systems, further reported that lentil hull extracts and their digestive products attenuated inflammatory responses and were linked to barrier-related effects [71]. This interpretation is further supported by Guo et al. (2023), who showed that lentil hull polyphenols are bioaccessible, selected digestion-released compounds can cross Caco-2 monolayers, and their *in vivo* metabolism involves extensive phase I/II biotransformations [70]. Taken together, these findings indicate that lentils provide both fermentable starch residues and digestion-transformed phenolic pools that remain biologically relevant at the epithelial interface, although their combined effects have not yet been directly tested in lentil-specific interaction studies.

From a broader mechanistic perspective, the complementarity between resistant starch and phenolics is biologically coherent. Resistant starch primarily influences gut physiology through microbial fermentation and the generation of short-chain fatty acids, especially butyrate, which serves as a major fuel for colonocytes and is linked to epithelial differentiation, mucus-associated defense, tight junction maintenance, and restraint of inflammatory signaling [108,110,113]. Polyphenols, by contrast, contribute less as major energetic substrates and more as regulatory compounds whose digestion products and microbial metabolites can influence inflammatory tone, oxidative balance, host–microbe signaling, and the ecological behavior of the gut microbiota [72,118]. Because these two routes act on overlapping aspects of mucosal homeostasis, their actions need not be identical to be functionally compatible. Resistant starch sustains metabolic support through SCFAs, while polyphenols help constrain inflammatory perturbations. Thus, the most evidence-consistent interpretation is that, within the lentil matrix, resistant starch and phenolics provide complementary inputs to gut barrier maintenance, with current evidence supporting convergence and plausible reinforcement more strongly than directly demonstrated synergy.

Taken together, the co-localization of RS and polyphenols within lentil matrices, combined with their shared reliance on microbial processing, suggests that lentils provide a dietary environment conducive to barrier preservation through multiple complementary mechanisms.

Table 3 provides an overview of representative lentil bioactives across different groups, together with the experimental models used, the main mechanisms reported, and their gut health-related outcomes.

## 6. Potential Complementary Interactions Among Lentil Bioactives 

### 6.1. Interplay Between Peptides, Resistant Starch, and Polyphenols During Digestion

A central but often under-appreciated premise for potential interplay among lentil peptides, resistant starch (RS), and polyphenols is that their functional bioactivity is largely determined upstream by matrix-governed transformations during digestion, which dictate (i) what chemical species are released, (ii) where along the gastrointestinal tract they become available, and (iii) the extent to which they reach the colon as fermentable/transformable substrates. Accumulating evidence suggests that their physiological relevance can be better understood within the context of digestive co-release and complementary interactions rather than any single compound acting alone.

Within lentils, matrix architecture already appears to be a key determinant of what becomes available during digestion. Duijsens et al. (2023) [30] showed that cooking time altered the *in vitro* amylolysis and proteolysis kinetics of whole cooked lentils, with shorter cooking preserving more cell clusters and slowing starch digestion relative to more extensively cooked samples. Duijsens et al. (2024) [123] further demonstrated that moving from static to semi-dynamic digestion conditions relevant to older adults changed starch and protein digestion trajectories in cooked lentils. Together, these studies indicate that lentil digestion is governed not simply by composition, but by cell integrity, microstructure, and gastrointestinal conditions, all of which are likely to influence the relative release of peptides, the persistence of starch-derived residues, and the exposure of polyphenol-rich outer tissues during digestion [30,123].

Direct evidence that protein and phenolic fractions can influence one another during lentil digestion is also beginning to emerge. Boachie et al. (2022) showed that interaction between lentil proteins and tannic acid induced aggregation, limited peptic hydrolysis, and altered the resulting peptidomic profile, indicating that polyphenol binding can reshape peptide release in a lentil system rather than only in model proteins [124]. In parallel, Guo et al. (2022) demonstrated that digestive products derived from free and bound lentil hull phenolics retained anti-inflammatory activity in HT-29 cells and were associated with coordinated modulation of NF-κB and Keap1–Nrf2 signaling [47]. Peng et al. (2022), using Caco-2 monolayers and Caco-2/RAW264.7 co-culture systems, further reported that lentil hull digestive products attenuated inflammatory responses and supported barrier-relevant outcomes [71]. This evidence is extended by Guo et al. (2023), who showed that lentil hull polyphenols are bioaccessible, selected digestion-released compounds can cross Caco-2 monolayers, and subsequent in vivo metabolism involves extensive phase I/II biotransformations [70]. Taken together, these studies support a digestion-dependent narrowing of the biologically relevant lentil bioactive pool to transportable and transformed entities, although they do not yet resolve how co-digested peptides and starch jointly modulate these outcomes in a true ternary design.

On the starch side, directly relevant lentil evidence is now available for the downstream consequence of digestion- and processing-conditioned structure. Zhao et al. (2024) [27] showed that resistant starch derived from untreated and autoclaved lentil starch followed distinct multiscale structural trajectories during in vitro colonic fermentation and differentially regulated microbiota composition. Although this study begins from resistant starch preparations and colonic fermentation rather than upper gastrointestinal co-digestion itself, it strengthens a key inference for the present section: the structural state in which lentil starch enters the colon is not fixed, and digestion or processing history is likely to condition both fermentability and interaction with co-delivered bioactives [27].

Broader food-model studies help explain why binary and ternary interactions during digestion merit attention, but they are best treated as mechanistic background rather than lentil evidence. Hamzalioglu et al. (2023) showed in a casein–phenol model that digestive conditions can generate protein–phenol interactions that alter both peptide and phenol bioaccessibility [125]. Likewise, Meng et al. (2025) demonstrated in a rice starch–polyphenol–whey protein isolate system that the presence of protein substantially changes how polyphenols affect starch structure and digestibility, largely by competing for polyphenol binding and modifying the contribution of amylase inhibition relative to structural ordering [126]. These studies do not establish dietary equivalence to lentils, but they do show that digestion-phase interactions in complex food matrices are mechanistically plausible and can materially alter downstream substrate delivery.

Overall, current evidence supports a digestion-centered model in which lentil microstructure governs macronutrient release, protein–polyphenol interactions can reshape peptide liberation, digestion transforms hull-associated phenolics into bioaccessible and transportable derivatives, and the structural state of starch conditions later fermentation behavior. What remains unproven is whether these processes interact strongly enough in lentils to generate a measurable ternary interaction effect. For that reason, this subsection supports digestion-phase complementarity more strongly than directly demonstrated synergy. Future lentil studies would benefit from multi-component designs embedded in standardized workflows such as INFOGEST, so that results become more comparable across model systems and mechanistic endpoints [27,30,70,93].

### 6.2. Co-Regulation of Microbiota Composition and Metabolic Pathways

Microbiome-level coordination among lentil-derived bioactives is best interpreted through changes in substrate availability, metabolic routing, and cross-feeding as microbiota responses are highly contingent on baseline community structure, substrate hierarchy, and exposure time. In this context, the relevant evidence is stronger for coordinated shifts in microbial composition and metabolite output than for direct proof of epithelial barrier protection.

Existing evidence on lentils already indicates that whole lentil matrices can reshape both the composition and the activity of the colonic microbiota. In healthy male mice, Graf et al. (2019) [49] showed that diets supplemented with cooked red lentils increased microbial α-diversity, shifted community structure, and enriched SCFA-associated genera such as *Prevotella*, *Roseburia*, and *Dorea*, while also increasing total fecal SCFAs, including acetate and butyrate. Importantly, the study compared lentil-containing diets with both a basal diet and a pectin control, which supports a role for the integrated lentil matrix beyond a simple soluble-fiber explanation. This matters for the microbiota composition because it shows that lentils can modify microbial ecology and metabolite output as a whole food system, consistent with coordinated action among multiple substrate classes rather than a single isolated constituent [49]. For lentil starch, Zhao et al. (2024) [27] showed that resistant starch prepared from untreated versus autoclaved lentil starch underwent distinct structural trajectories during dynamic *in vitro* fermentation and differentially enriched microbial groups, with untreated lentil RS favoring early *Bifidobacterium* growth and autoclaved lentil RS enriching *Ruminococcus*. This finding is mechanistically important because it links microbiota composition not simply to “more RS,” but to the structural state in which lentil starch reaches the colon. In other words, lentil starch appears to regulate microbial metabolism through a structure-dependent hierarchy of accessibility: primary degraders respond first to the architecture of the residual glucan, and the resulting intermediates then become available to additional taxa through cross-feeding, thereby shaping downstream metabolite profiles as well as community succession [27].

Although direct evidence for lentil proteins remains less developed than that for starch and phenolics, it is beginning to emerge. Mastrolonardo et al. (2025) [52], using a SHIME^®^ dynamic gut model, reported that fermented red lentil protein isolate promoted the growth of potentially beneficial genera such as *Lactiplantibacillus* and *Furfurilactobacillus*, increased SCFA production with a notable rise in butyrate, and generated a greater release of low-molecular-weight peptides than the raw isolate. These data are still model-specific and do not establish how protein-derived peptides behave within intact lentil matrices, but they do suggest that lentil protein processing can change the form in which nitrogenous substrates and bioactive peptides enter the colonic ecosystem, with consequences for both microbial composition and metabolic output. This is relevant because proteolytic substrates do not act independently of saccharolytic ones; rather, their effects depend on whether fermentable carbohydrates and polyphenols are simultaneously present and able to redirect microbial metabolism away from more detrimental proteolytic end products [52].

For phenolics, the most direct lentil evidence here lies in showing that these compounds can reach the colon as transformed yet biologically relevant substrates. Mustafa et al. (2022) [5] summarized that lentil phenolics are concentrated predominantly in the hull fraction, while Guo et al. (2023) [70] showed that simulated digestion progressively released lentil hull polyphenols, selected compounds could cross Caco-2 monolayers, and *in vivo* metabolism involved extensive hydroxylation, methylation, glucuronidation, and sulfation. These observations do not by themselves define the microbiota response, but they establish a necessary precondition for it: lentil phenolics do not simply disappear during digestion, but arrive downstream as transformed substrates that are available for further microbial conversion [5,70].

Pulse-level evidence reinforces this interpretation. In an aged humanized murine model, Kadyan et al. (2023) [107] showed that resistant starches derived from dietary pulses, including lentils, generated distinct microbiome–metabolome signatures, with metabolite–taxon associations varying by substrate source and host context. In the lentil arm, the metabolomic profile differed from control and was associated with increased abundance of metabolites such as acetate among a broader set of treatment-responsive compounds, while multi-omics integration linked beneficial metabolites with taxa including lactobacilli-related groups, *Bacteroides*, *Parabacteroides*, and *Parasutterella*. The key implication is not that lentil RS alone has already been mapped to a single conserved microbial signature, but that pulse-derived RS can shift microbial metabolism toward distinct functional states depending on substrate structure and host context [107].

Beyond lentils specifically, controlled fermentation and mechanistic syntheses help explain how polyphenols and fermentable carbohydrates can co-regulate microbial ecology. In an *in vitro* fermentation model inoculated with pooled healthy-donor stool, Whitman et al. (2024) [127] showed that a polyphenol blend increased absolute abundance of taxa such as *Ruminococcus bromii*, *Bifidobacterium*, and *Lactobacillus*, and that combining polyphenols with fermentable fiber produced microbial trajectories distinct from either component alone. This study was not a lentil model, but it provides proof-of-principle that polyphenols and fermentable carbohydrates can jointly reshape ecological niches rather than acting as isolated inputs [127]. Mechanistically, Alqudah and Claesen (2024) describe gut polyphenol metabolism as a scaffold-dependent sequence of hydrolysis, reduction, dehydroxylation, ring cleavage, and related reactions that generate smaller metabolites with altered biological activity and altered accessibility to different taxa [72]. In parallel, Le et al. (2024) argue that dietary fiber and (poly)phenols from bean systems frequently reach the colon together, where phenolic metabolites and fiber-derived SCFAs can jointly influence microbiota composition and host physiology, thus reinforcing the importance of matrix effects in shaping downstream microbial metabolism [128]. Taken together, these data support a model in which phenolics help shape microbial niche occupancy, while resistant carbohydrate helps determine which saccharolytic and cross-feeding pathways can be sustained over time.

Protein-derived regulation should still be interpreted more cautiously than the starch and phenolic branches. Reviews on protein–microbiota interactions show that peptides and amino acid residues reaching the colon can serve as nitrogenous substrates for microbial growth, but the resulting metabolic profile is highly dependent on the simultaneous availability of fermentable carbohydrate [129,130]. When saccharolytic substrates are abundant, microbial communities are more likely to channel metabolism toward SCFA production and away from the accumulation of potentially harmful proteolytic metabolites; when they are scarce, proteolytic routing becomes more prominent [131,132]. In lentil-rich systems, this suggests that peptides are unlikely to act as fully independent regulators of the microbiota. Rather, they should be viewed as part of a substrate network in which nitrogen availability, RS accessibility, and phenolic metabolism are interdependent determinants of microbial composition and function.

Overall, the current evidence supports coordinated microbiome and metabolome responses to lentil-relevant substrates. Lentil and pulse studies already show that whole lentils, lentil RS, and processed lentil protein fractions can alter microbial composition and SCFA-related outputs, while lentil phenolic studies establish that transformed phenolic substrates remain available for downstream microbial metabolism. More general fermentation and mechanistic studies then help explain how these substrates may converge: resistant starch shapes carbohydrate accessibility and cross-feeding, phenolics bias ecological filtering and microbial biotransformation, and peptides contribute conditional nitrogen inputs that can modify metabolic routing [27,49,52,70,72,127,128].

### 6.3. Conceptual Model of Lentil Bioactives in Gut Health

Under a strict biological definition, synergistic protection of intestinal barrier integrity would require two or more bioactive components to act simultaneously and produce effects that cannot be attributed to either component alone. In lentils, direct evidence meeting this criterion remains limited. The current literature more strongly supports a coordinated framework in which lentil-derived peptides, resistant starch, and polyphenols contribute through partially distinct but biologically compatible routes that intersect during digestion, colonic co-metabolism, and downstream host signaling.

At the digestion stage, the strongest lentil evidence concerns matrix-dependent release and transformation of bioactives. Boachie et al. (2022) [124] showed that tannic acid induced aggregation of lentil proteins, reduced peptic hydrolysis and altered the resulting peptidomic profile, indicating that protein–polyphenol interaction can modify peptide liberation in a lentil system. Guo et al. (2022) [47] showed that digestive products derived from free and bound lentil hull phenolics retained anti-inflammatory activity in HT-29 cells and were linked to coordinated modulation of NF-κB and Keap1–Nrf2 signaling. Peng et al. (2022) [71], using Caco-2 monolayers and Caco-2/RAW264.7 co-culture systems, further reported that lentil hull digestive products attenuated inflammatory responses and supported barrier-relevant outcomes. Guo et al. (2023) [70] extended this picture by showing that lentil hull polyphenols are bioaccessible, selected digestion-released compounds can traverse Caco-2 monolayers, and subsequent *in vivo* metabolism involves extensive phase I/II biotransformations. On the starch side, Zhao et al. (2024) [27] showed that resistant starch prepared from untreated and autoclaved lentil starch followed distinct structural trajectories during dynamic *in vitro* fermentation and differentially regulated the microbiota. Taken together, these studies support a digestion-dependent narrowing of the lentil bioactive pool to transformed phenolic derivatives, fermentation-active starch residues, and peptide mixtures whose release can be modified by polyphenol binding [27,47,70,71,124].

Once these substrates reach the colon, the evidence is stronger for coordinated effects on microbial composition and metabolic routing than for direct multi-component synergy. Graf et al. (2019) [49] showed that diets supplemented with cooked red lentils altered the colonic microenvironment in mice, including shifts in community structure and increased fecal short-chain fatty acids. At the pulse level, Kadyan et al. (2023) [107] showed that resistant starches derived from dietary pulses, including lentils, generated distinct microbiome–metabolome signatures in aged, humanized mice rather than a uniform fermentation response. The protein branch remains less developed than the starch and phenolic branches, but it is no longer absent: Mastrolonardo et al. (2025) [52] reported in a SHIME^®^ dynamic gut model that fermented red lentil protein isolate increased the release of low-molecular-weight peptides, promoted several potentially beneficial genera, and increased SCFA production, particularly butyrate. Together, these studies indicate that lentil-relevant substrates can reshape both microbial composition and metabolic output, although they do not yet demonstrate that peptides, RS, and phenolics interact synergistically in intact lentil matrices [49,52,107].

The phenolic branch contributes a different but compatible layer of regulation. Mustafa et al. (2022) [5] summarized that lentil polyphenols are concentrated predominantly in the hull fraction, while Guo et al. (2023) [70] showed that these compounds survive digestion in transformed form and remain available for downstream transport and metabolism. More broadly, Alqudah and Claesen (2024) [72] describe gut polyphenol metabolism as a scaffold-dependent sequence of deglycosylation, reduction, dehydroxylation, and ring cleavage that generate smaller metabolites with altered biological activity and accessibility to different taxa. In parallel, Whitman et al. (2024) [127] showed in a non-lentil *in vitro* fermentation model that polyphenol–fiber blends produced microbial trajectories distinct from either component alone, while Le et al. (2024) [128] argued from bean systems that dietary fiber and (poly)phenols commonly reach the colon together and undergo coupled microbial metabolism. Within the lentil framework, the most defensible interpretation is therefore that phenolics contribute less as major energetic substrates and more as regulatory substrates that influence microbial niche occupancy, pathway activation, and inflammatory tone [5,70,72,127].

Reviews on protein–microbiota interactions indicate that peptides and amino acid residues reaching the colon can serve as nitrogenous substrates for microbial growth, but the resulting metabolic profile depends strongly on the concurrent availability of fermentable carbohydrate. When saccharolytic substrates are abundant, microbial metabolism is more likely to be directed toward SCFA production and away from the accumulation of less desirable proteolytic end products; when they are limited, proteolytic routing becomes more prominent. In lentil-rich systems, this suggests that peptides are unlikely to function as fully independent regulators of the microbiota. Rather, they are better viewed as part of a substrate network in which nitrogen availability, RS accessibility, and phenolic metabolism jointly shape microbial composition and function [129,130,131,132].

Within this framework, resistant starch, peptides, and phenolics need not exert identical functions to act in a coordinated manner. Resistant starch primarily contributes fermentable carbon that supports saccharolytic fermentation and SCFA generation; peptides and amino acid residues provide conditional nitrogen inputs whose effects depend on the concurrent availability of fermentable carbohydrate; and phenolics, together with their microbial metabolites, help shape microbial niche occupancy and substrate selectivity. Their convergence is therefore best understood as a matrix-conditioned form of complementarity in which substrate form, release timing, and co-metabolism collectively influence the metabolic environment to which the intestinal epithelium is exposed. This interpretation is consistent with the current evidence base and does not require assuming that true ternary synergy has already been demonstrated in lentils [27,47,48,49,52,70,72,124].

**Figure 2:**
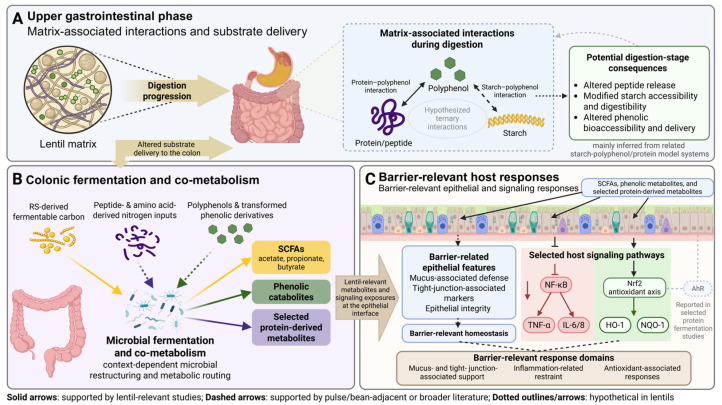
Evidence-weighted framework of complementary interactions among lentil bioactives linking digestion, colonic co-metabolism, and barrier-relevant host responses.

As summarized in Figure 2, current evidence supports a matrix-conditioned and evidence-weighted framework in which lentil-derived peptides, resistant starch, and polyphenols contribute to gut homeostasis through complementary routes spanning digestion, colonic co-metabolism, and barrier-relevant host responses.

## 7. Gut Barrier Mechanisms and Evidence

### 7.1. Molecular Pathways in Intestinal Protection

Available evidence suggests that lentil-associated preservation of intestinal barrier integrity involves a limited number of recurring molecular control nodes, although the strength of direct evidence differs across pathways. At present, the clearest lentil-specific pathway-level evidence is derived from phenolic-rich hull fractions and their digestive products, which have been linked to modulation of inflammatory signaling, redox homeostasis, and tight junction-associated barrier responses in intestinal cell-based systems. By comparison, fermentation-linked SCFA receptor signaling represents a biologically plausible barrier-relevant pathway in lentil research, but part of its downstream mechanistic interpretation is informed by broader non-lentil literature rather than being fully resolved in lentil-specific models.

Among the currently available studies, lentil hull-derived phenolic fractions provide the most detailed evidence for direct modulation of barrier-relevant epithelial signaling. In a HT-29 intestinal epithelial model, Guo et al. (2022) [47] examined the anti-inflammatory mechanism of laird lentil hull digestive products based on the NF-κB and Keap1-Nrf2 signaling pathways. The study reported that lentil hull phenolic digestive products suppress inflammation in HT-29 cells through the inhibition of NF-κB signaling and activation of the Keap1–Nrf2 antioxidant defense pathway. Under inflammatory stimulation, lentil hull digestive products markedly suppressed the activation of the NF-κB pathway, as indicated by a reduction in IκBα phosphorylation and degradation, which in turn limited the nuclear translocation of the NF-κB p65 subunit. Consistent with the inhibition of NF-κB signaling, the expression of key pro-inflammatory mediators, including IL-8 and IL-6, was significantly decreased at both transcriptional and protein levels. In parallel, lentil hull digestive products significantly activated the Keap1–Nrf2 pathway, as evidenced by enhanced dissociation of Nrf2 from Keap1, increased nuclear accumulation of Nrf2, and the subsequent upregulation of downstream antioxidant and cytoprotective enzymes, particularly HO-1 and NQO-1. The activation of Nrf2 signaling was accompanied by a pronounced reduction in intracellular reactive oxygen species, indicating attenuation of oxidative stress in inflamed HT-29 cells. Notably, digestive products derived from bound phenolics exhibited stronger regulatory effects on Nrf2 activation and HO-1 expression than those from free phenolics. Furthermore, the suppression of oxidative stress was associated with reduced NF-κB signaling intensity, suggesting that lentil hull digestive products modulated inflammatory responses through coordinated regulation of oxidative stress and inflammatory signaling [47]. Peng et al. (2022) [71] examined the effect of lentil hull extract via MAPK/NF-κB signaling pathways and the effect of digestive products on intestinal barrier and inflammation in Caco-2 and Raw264.7 co-culture. The results showed that lentil hull extracts suppressed macrophage-mediated inflammatory signaling by inhibiting MAPK (ERK, JNK, p38) phosphorylation and subsequent NF-κB activation, leading to the reduced production of NO, TNF-α, and IL-6. This immune modulation attenuated the inflammatory pressure exerted on intestinal epithelial cells. Meanwhile, digestive products of lentil hull directly act on intestinal epithelial cells to restore tight junction protein expression and localization, including ZO-1 and occludin, thereby reducing epithelial permeability under inflammatory conditions. Through the coordinated regulation of immune cell activation and epithelial barrier structure, lentil hull-derived bioactives effectively preserve intestinal barrier integrity [71]. Together, these studies provide lentil-specific evidence suggesting that lentil hull-derived phenolics exert intestinal protective effects through the coordinated regulation of inflammatory signaling and epithelial barrier integrity, integrating macrophage-mediated immune modulation, digestion-enhanced bioactivity, and direct stabilization of intestinal tight junctions rather than by targeting a single downstream effector.

Additional lentil-specific support is provided by studies focusing on epithelial-intrinsic inflammatory control. In an epithelial-focused model, Maqoud et al. (2024) [133] evaluated a novel acetonitrile–water extract (AWE) of *Lens culinaris* for its protective effects against lipopolysaccharide (LPS)-induced inflammatory damage in Caco-2 intestinal epithelial cells, providing mechanistic insight into how lentil-derived bioactives modulate molecular pathways within the intestinal epithelium itself. LPS stimulation triggered a pronounced inflammatory response in Caco-2 cells, characterized by the activation of both the nuclear factor-κB (NF-κB) and mitogen-activated protein kinase (MAPK) signaling cascades, including the phosphorylation of IκBα, nuclear translocation of NF-κB p65, and enhanced activation of the three major MAPK branches—extracellular signal-regulated kinase (ERK1/2), c-Jun N-terminal kinase (JNK), and p38 MAPK. These signaling events collectively promoted the upregulation of epithelial pro-inflammatory mediators, notably interleukin-8 (IL-8) and interleukin-6 (IL-6), as well as inflammation-associated enzymes such as cyclooxygenase-2 (COX-2) and inducible nitric oxide synthase (iNOS), leading to inflammation-related epithelial injury and reduced cell viability. Treatment with the lentil-derived AWE markedly attenuated this epithelial inflammatory phenotype through the coordinated inhibition of NF-κB and MAPK signaling pathways. At the level of NF-κB activation, AWE suppressed IκBα phosphorylation and degradation, thereby limiting the nuclear translocation of NF-κB p65 and reducing the downstream transcriptional activation of pro-inflammatory genes. In parallel, AWE exerted a broad inhibitory effect on MAPK signaling, significantly decreasing the phosphorylation of ERK1/2, JNK, and p38, indicating that the extract acts upstream to inhibit multiple converging inflammatory inputs rather than targeting a single downstream effector. The simultaneous suppression of these pathways resulted in a substantial reduction in epithelial-derived inflammatory mediators, including IL-8 and IL-6, as well as decreased expression of COX-2 and iNOS, ultimately mitigating LPS-induced epithelial damage and improving cell viability [133]. From a molecular pathway perspective, this study further supports a barrier-relevant protective interpretation by showing the direct modulation of epithelial inflammatory signaling. By interrupting the MAPK–NF-κB signaling axis at multiple regulatory nodes, the acetonitrile–water extract may help prevent the establishment of a self-amplifying inflammatory loop within intestinal epithelial cells. These findings complement immune-centric models of gut protection and underscore the importance of epithelial-intrinsic pathway modulation as a critical component of intestinal defense, highlighting NF-κB and MAPK signaling pathways as central molecular targets through which lentil-derived phenolics contribute to intestinal homeostasis.

Panaro et al. (2024) [101] provided supplementary but mechanistically relevant lentil-specific evidence by linking epithelial anti-inflammatory activity with *ex vivo* intestinal spasmolytic effects in a study explicitly designed around IBD-related inflammation and altered motility. Using phenolic-rich extracts derived from lentil seed coats, the epithelial inflammatory responses were examined in an LPS-stimulated Caco-2 intestinal epithelial cell model, which mimics innate immune activation associated with IBD. In this model, lentil waste extracts markedly downregulated Toll-like receptor 4 (TLR4) protein expression, indicating the attenuation of an upstream pathogen-recognition pathway at the epithelial level. This was accompanied by the reduced transcription of inducible nitric oxide synthase (iNOS), which is a key mediator of nitrosative stress and epithelial injury, as well as a coordinated shift in cytokine expression characterized by decreased IL-1β and increased IL-10 levels. These changes support the interpretation that lentil-derived phenolic extracts can suppress epithelial inflammatory activation and promote a more anti-inflammatory epithelial signaling profile. Although these readouts do not on their own establish a fully resolved downstream signaling cascade, they are consistent with the attenuation of upstream pro-inflammatory signaling relevant to intestinal epithelial stress. In addition to these epithelial findings, this study examined functional responses in *ex vivo* guinea pig ileal and proximal colonic preparations and reported pronounced spasmolytic effects of lentil waste extracts. These extracts reduced spontaneous smooth muscle contractility in both longitudinal and circular muscle layers, attenuated indices of excessive basal tone, and modulated contractility-related indices linked to transit speed, pain, mixing, and fragmentation. From a physiological perspective, these observations are relevant because abnormal intestinal spasm and dysregulated motor activity frequently accompany inflammatory bowel disease and contribute to symptom burden. Accordingly, the *ex vivo* data suggest that lentil-derived phenolic fractions may provide functional benefit not only through attenuation of epithelial inflammatory stress but also through the modulation of inflammation-associated intestinal motility disturbances. At the same time, the spasmolytic findings should be interpreted as functionally relevant support for intestinal symptom control rather than as direct evidence of epithelial barrier regulation [101]. Overall, this study extends the mechanistic scope of lentil phenolic research by indicating that phenolic-rich lentil waste extracts may act at both the epithelial inflammatory level and the level of gut motor function, while the direct barrier-related interpretation remains primarily anchored in the epithelial anti-inflammatory data rather than in the ex vivo motility assays.

A further pathway relevant to lentil-associated intestinal protection is the fermentation-linked SCFA–GPCR axis. Lentil is rich in microbiota-accessible carbohydrates, including resistant starch and fermentable dietary fiber, which can resist digestion in the upper gastrointestinal tract and undergo colonic fermentation. In a well-controlled *in vivo* study, dietary supplementation with cooked red lentils in healthy C57BL/6 male mice significantly increased fecal short-chain fatty acid (SCFA) concentrations and altered the colonic microbial environment. Importantly, lentil feeding was associated with upregulated colonic expression of SCFA-sensing G protein-coupled receptors (GPCRs), specifically Gpr41/FFAR3 and Gpr43/FFAR2, accompanied by an increased mRNA expression of SCFA receptors (Gpr41/Gpr43/Gpr109a) and junction/adhesion markers (ZO-1, claudin-2, E-cadherin). These coordinated molecular changes indicate that lentil-derived fermentation products promote an SCFA-responsive mucosa and support transcriptional programs linked to epithelial barrier integrity, although the authors noted that functional permeability assays are required to confirm improvements in barrier function [49]. The mechanistic basis for this receptor-mediated sensing is well-established. Brown (2003) [134] demonstrated that propionate and other short-chain carboxylic acids act as endogenous ligands for GPR41 and GPR43, confirming these GPCRs as bona fide receptors for microbial fermentation metabolites. This ligand–receptor interaction provides a biologically plausible route through which lentil-derived SCFAs may influence host epithelial and immune signaling pathways relevant to mucosal homeostasis [134]. From a mucosal immunology perspective, intestinal protection is inherently a coupled process, integrating the preservation of epithelial barrier integrity with the suppression of excessive mucosal inflammation. Evidence from non-lentil-specific but mechanistically relevant studies indicates that SCFA–GPCR signaling contributes to both arms of this protection. For example, the activation of GPR109A/HCAR2—a receptor responsive to butyrate—was shown to be essential for the butyrate-mediated induction of epithelial IL-18, maintenance of epithelial integrity, and protection against colonic inflammation and carcinogenesis *in vivo*. This work links microbial metabolites to epithelial-derived protective cytokine signaling, underscoring how anti-inflammatory effects are embedded within barrier-protective mechanisms rather than acting independently [135]. In parallel, signaling through FFAR2/GPR43 has been identified as a critical mediator of dietary fiber-induced intestinal protection. Genetic and dietary studies demonstrated that FFAR2 deficiency abolished the beneficial effects of fiber-derived SCFAs on gut microbial composition and susceptibility to intestinal pathology, highlighting the importance of SCFA–GPCR signaling in maintaining mucosal homeostasis. While such downstream mechanisms have not been universally validated using lentils specifically, the convergence of lentil-induced SCFA production, upregulation of SCFA-sensing GPCRs, and established GPCR-dependent barrier and anti-inflammatory pathways supports the biological plausibility of an SCFA–GPCR-mediated contribution to lentil-associated intestinal protection, while acknowledging that several downstream steps are inferred from non-lentil mechanistic studies [136].

An important strength of these studies is that many of them preserve, at least in part, the physiological matrix in which lentil bioactives are delivered, thereby capturing interactions among phenolics, fermentable carbohydrates, and protein-associated components. At the same time, this creates an attribution challenge: matrix-level efficacy does not identify which fraction is necessary, sufficient, or dominant, nor does it determine whether the observed barrier-relevant effects reflect complementary actions within the food matrix or true synergy. This limitation should be kept in mind when interpreting mechanistic convergence across lentil studies.

### 7.2. In Vitro, Animal, and Limited Clinical Evidence Supporting Lentil Bioactives and Gut Health

The available evidence spans *in vitro* epithelial systems, *in vitro* fermentation platforms, animal models, and a limited number of human intervention studies. These models address different biological questions and are not directly equivalent. *In vitro* epithelial studies primarily inform inflammatory signaling, cytoprotection, and selected barrier-associated readouts, whereas fermentation systems mainly capture microbial metabolism and fermentation-derived metabolite profiles. Animal studies provide more integrative evidence by combining inflammatory, histological, permeability, and sometimes microbiome-related outcomes, while current human studies mainly support feasibility, tolerability, and microbiome or metabolic relevance rather than direct confirmation of barrier protection.

*In vitro* evidence for lentil-linked barrier effects is strongest when studies quantify epithelial integrity endpoints and pair them with inflammatory readouts. Peng et al. (2022) [71] combined simulated digestion with epithelial models and reported barrier protection signals in Caco-2 systems along with reduced inflammatory activation in their co-culture design, providing a mechanistically informative bridge between digestion chemistry and epithelial outcomes. In this study, researchers used *in vitro* digestion together with Caco-2 monoculture and Caco-2/RAW264.7 co-culture models. Their primary outcomes included NO, IL-6, IL-1β, iNOS/COX-2, and MAPK/NF-κB signaling, together with barrier-associated endpoints such as fluorescein sodium permeability (Papp), claudin-1, and ZO-1, making this one of the clearest lentil-focused *in vitro* studies linking inflammatory regulation with functional barrier-related readouts [71].

Using HT-2 cells, Guo et al. (2022) [47] demonstrated that digestive products derived from lentil hull phenolics retained anti-inflammatory activity through NF-κB inhibition and Keap1–Nrf2 activation, supporting the premise that digestion-transformed lentil components can remain bioactive at the intestinal epithelium. This study is more appropriately interpreted as pathway-resolved anti-inflammatory evidence than as direct functional confirmation of barrier restoration, because it relies on a cancer cell line and signaling-dominant outcomes rather than permeability-based endpoints [47]. Beyond this, Panaro et al. (2024) [101] evaluated lentil waste or hull extracts in inflammation-relevant intestinal cell settings and discussed their potential value for IBD-oriented nutrition strategies, adding further *in vitro* support for lentil by-product bioactivity in gut-related endpoints. Combining LPS-stimulated Caco-2 cells with *ex vivo* ileal and colonic contractility assays, this research showed that lentil waste or hull extracts modulated inflammatory markers such as iNOS, IL-1β, and IL-10 together with spasmolytic effects. These findings support anti-inflammatory and motility-related relevance, but they should be interpreted as indirect barrier-supportive evidence rather than direct proof of epithelial barrier restoration [101]. Maqoud et al. (2024) [133] further contributed *in vitro* evidence by testing a *Lens culinaris* extract in LPS-challenged Caco-2 cells with outcomes described as the protection of intestinal mucosal integrity in that experimental context. Importantly, this study also reported improved transepithelial electrical resistance (TEER) and tight junction protein levels together with reduced TNF-α, NF-κB, IL-1β, and IL-8, indicating that lentil extract can preserve epithelial barrier-associated function under inflammatory challenge; nevertheless, it remains an epithelial mono-culture model lacking immune and microbiome components [133].

Animal trial evidence provides more integrative endpoints, including histology, inflammatory markers, and barrier-associated protein. In a DSS-induced colitis mouse model, Graf et al. (2020) [50] reported that red lentil pre-feeding reduced colitis severity and increased colon barrier function, supporting a protective effect of whole red lentil supplementation in an inflammatory model. More specifically, male C57BL/6 mice were pre-fed a basal diet or an isocaloric 20% red lentil-supplemented diet for 3 weeks before acute colitis was induced with 2% DSS in drinking water for 5 days; indexed records indicate n = 12 per dietary group/time point. Primary outcomes included clinical disease severity, colon histology, pro-inflammatory cytokines, and barrier-/repair-related markers such as IL-22, Relmβ, occludin, and serum lipopolysaccharide-binding protein [50]. More recently, Chen et al. (2025) [100] evaluated cellulase-modified lentil hull soluble dietary fiber (SDFM) in a DSS-induced colitis and behavioral-deficit model using 48 male C57BL/6J mice (6–8 weeks old; n = 12/group) assigned to CON, DSS, LSDF (500 mg/kg/day), or HSDF (1000 mg/kg/day). The study integrated DAI, colon length, colon and brain histopathology, serum LPS and BDNF, RT-qPCR, 16S rRNA sequencing, fecal SCFAs, and brain metabolomics, and reported that SDFM significantly improved both colitis-related and behavioral outcomes, with LSDF generally outperforming HSDF. The intervention was also associated with shifts in harmful and beneficial bacterial taxa and with alterations in sphingolipid-related brain metabolites, supporting a gut–brain-axis mechanism [100]. In addition to inflammatory models, Graf et al. (2019) provided complementary evidence from a healthy feeding study on C57BL/6 male mice (n = 12/group), in which diets supplemented with 5%, 10%, or 20% cooked red lentils by weight, together with a 0.7% pectin comparator, were used to assess colonic microenvironment outcomes including microbiota activity and epithelial barrier integrity/function [49]. Likewise, Kadyan et al. (2024) [111] examined lentil-resistant starch in a humanized murine model of aging: 60-week-old C57BL/6J mice colonized with human microbiota were fed a western-style high-fat diet for 20 weeks, with lentil-resistant starch incorporated at 5% *w*/*w*; the group size was n = 14–16/group (7–8 per sex). Endpoints included microbiome shifts, gut hyperpermeability, and tight junction-related markers, supporting a microbiota-dependent route to barrier preservation [111]. Although these animal studies are more physiologically integrated than cell systems, direct comparison across them remains limited by differences in host background, sex distribution, model induction, intervention format, and endpoint selection. In addition, acute chemically induced colitis models and purified-fraction interventions do not fully recapitulate chronic human dietary exposure or disease complexity.

Human evidence remains comparatively sparse with respect to direct barrier endpoints, but recent trials strengthen the translational plausibility of lentil-based interventions. Chamberlin et al. (2024) [103] conducted a randomized clinical trial of daily lentil consumption. This trial enrolled 38 overweight and obese adults and compared 0 versus 980 g of cooked whole green lentils per week provided as a mid-day meal for 12 weeks. It reported improvements in metabolic and inflammatory responses without gastrointestinal adverse effects, establishing feasibility and systemic anti-inflammatory signals in humans that are consistent with gut-mediated mechanisms [103]. A separate randomized clinical trial studied by Wilson et al. (2022) [137] examined metabolically at-risk, non-diabetic adults in an 8-week intervention using five prepared mid-day meals per week containing 0 g, 300 g, or 600 g cooked green lentils weekly; 30 adults completed the intervention. It reported a reduction in the progression of insulin resistance without increasing the severity of gastrointestinal symptoms, supporting the idea that a sustained intake of lentils is tolerable and metabolism-related. The primary outcomes of this study focused on glycemic control, HOMA-IR, and gastrointestinal symptoms rather than direct barrier-specific measures [137].

Beyond metabolic endpoints, Wilson et al. (2025) [138] conducted a 12-week parallel randomized controlled trial on metabolically at-risk adults (n = 36) with elevated waist circumference (≥102 cm, males; ≥88 cm, females) and triglycerides (≥1.7 mmol/L), comparing mid-day meals containing 0 or 980 g/week of whole green lentils. Using pre- and post-intervention fecal samples for 16S rRNA sequencing, the study showed that lentil consumption altered gut microbial composition at the family (*p* = 0.03) and OTU level (*p* = 0.01), with a borderline genus-level effect (*p* = 0.053), and increased the abundance of *Bifidobacterium*. These microbial shifts were further associated with changes in postprandial GM-CSF and glucose responses, supporting a plausible microbiota-mediated pathway linking lentil intake to host metabolic health, although direct barrier-specific endpoints were not measured [138]. However, current clinical studies do not directly quantify intestinal permeability, tight junction integrity, or *ex vivo* barrier function, and therefore provide supportive translational context rather than a direct confirmation of barrier protection in humans. For broader context, pulses-focused synthesis work emphasizes that dietary pulses can modulate gut microbiota composition and function and influence inflammatory outcomes, supporting the placement of lentils within an evidence-based dietary pattern for gut health [139]. Taken together, the available evidence is directionally supportive but methodologically heterogeneous. Pathway-resolved evidence is strongest in epithelial cell models, integrated barrier-related phenotypes are more apparent in animal studies, and current human trials remain largely indirect because direct permeability or tight junction outcomes are rarely assessed. Accordingly, claims regarding coordinated or synergistic gut barrier protection should be interpreted with caution unless supported by comparable endpoints across model systems. The major study-level design features, barrier-specific endpoints, microbiome readouts, and translational limitations are summarized in Table 4.

### 7.3. Comparative Analysis of Mechanisms Among Lentil Bioactives

Available evidence suggests that the barrier-relevant actions of lentil bioactives are not supported by a single, equally resolved mechanistic framework; rather, the depth and specificity of evidence differ substantially among phenolic-rich fractions, fermentation-linked carbohydrate fractions, and protein-derived components. Among these, phenolic-rich lentil fractions currently provide the clearest lentil-specific pathway-level evidence. Studies using lentil hull extracts or digestion-derived phenolic fractions in intestinal cell-based models have consistently linked these fractions to suppression of pro-inflammatory signaling, particularly through NF-κB- and MAPK-associated pathways, together with the activation of antioxidant defense responses such as Keap1–Nrf2. In parallel, these studies have also reported improvements in tight junction-related readouts under inflammatory challenge, supporting the interpretation that lentil phenolics can act directly on epithelial inflammatory and redox signaling in a manner relevant to barrier preservation. However, most of this evidence is derived from in vitro or digestion-coupled systems, and thus provides stronger support for pathway identification than for fully integrated barrier outcomes *in vivo* [47,71,133].

In contrast, evidence for lentil fiber- and resistant starch-related effects is currently stronger at the level of whole food or fiber-associated *in vivo* outcomes than at the level of directly resolved epithelial signaling. In healthy mice, cooked red lentils were shown to modify the colonic microenvironment, including increases in short-chain fatty acid production and upregulation of transcripts associated with SCFA-sensing and epithelial junctional integrity [49]. In a DSS-induced colitis model, red lentil supplementation reduced disease severity and improved colon barrier-related outcomes, while lentil hull soluble dietary fiber has more recently been reported to ameliorate colitis-associated phenotypes in mice [50,100]. These studies support a fermentation-linked and barrier-relevant mode of action for lentil carbohydrate fractions. Nevertheless, the mechanistic interpretation of these effects still relies in part on broader literature regarding resistant starch fermentation, SCFA generation, and microbiota-mediated mucosal regulation, rather than on fully resolved lentil-specific epithelial signaling studies [48]. Accordingly, current evidence supports the biological plausibility of carbohydrate-driven barrier protection in lentils, but does not yet establish a complete lentil-specific signaling sequence from fermentation substrate to epithelial response.

Protein- and peptide-related mechanisms remain the least directly characterized in the context of lentil-associated barrier protection. Although broader research indicates that dietary protein source, digestibility, and processing can influence microbial metabolism and nitrogen utilization in ways that may affect mucosal homeostasis, lentil-specific studies rarely isolate protein- or peptide-derived fractions and examine their effects using quantitative barrier endpoints. As a result, current interpretations of peptide-related contributions are largely contextual and remain less experimentally resolved than those for phenolic-rich fractions or fermentation-linked carbohydrate fractions [129,130].

Overall, the current literature is more consistent with complementary or convergent actions within the lentil food matrix than with demonstrated synergy. Phenolic-rich fractions provide the most clearly defined lentil-specific pathway evidence; fiber- and resistant starch-related effects are more consistently supported by *in vivo* and fermentation-linked outcomes, but remain less pathway-resolved; and protein-related mechanisms are still comparatively underdefined. Because lentils deliver these components within an integrated matrix and factorial studies remain scarce, the relative contribution of each fraction, and the extent to which their effects are additive, complementary, or truly synergistic, cannot yet be determined with confidence. Future studies should therefore combine fractionation or factorial designs with harmonized barrier-relevant endpoints, including tight junction protein localization and functional permeability assays, to distinguish matrix-level complementary effects from true synergy more rigorously [140,141].

**Figure 3:**
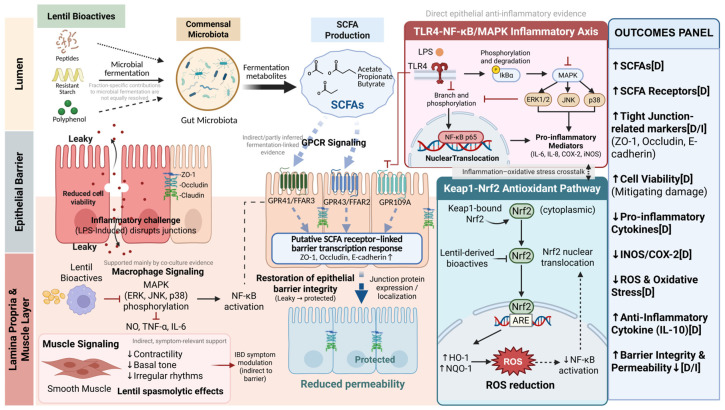
Evidence-weighted framework of cellular and microbial interactions relevant to lentil bioactives and intestinal barrier protection.

Figure 3 summarizes the current literature as an evidence-weighted framework, distinguishing direct lentil-specific evidence from indirect or partly inferred barrier-relevant interactions.

Phenolic-rich lentil fractions provide the clearest direct evidence for epithelial anti-inflammatory and antioxidant effects, including the modulation of TLR4/NF-κB/MAPK- and Keap1–Nrf2-associated pathways. Fermentation-linked carbohydrate fractions are associated with microbiota-derived SCFA production and receptor-related barrier responses, although several downstream steps remain partly inferred rather than fully resolved in lentil-specific systems. Protein- and peptide-related contributions remain comparatively underdefined. Solid links indicate direct lentil-specific evidence, whereas dashed links indicate indirect or lentil-relevant mechanistic support; dotted links denote hypothetical or unresolved interactions in lentils. Black arrows within pathway modules represent established signaling relationships and are not intended to indicate the strength of lentil-specific evidence. In the outcomes panel, ↑ indicates an increase, upregulation, or improvement, whereas ↓ indicates a decrease, downregulation, attenuation, or reduced impairment relative to the comparator condition. For combined entries, the arrows denote the direction of each respective outcome (e.g., ↑ barrier integrity and ↓ permeability). [D] indicates direct lentil-specific evidence, whereas [D/I] indicates outcomes supported by a combination of direct lentil-specific observations and indirect mechanistic inference. Accordingly, this figure should be interpreted as an evidence-weighted summary of direct, indirect, and putative barrier-relevant interactions rather than as a fully demonstrated lentil-specific mechanistic map.

## 8. Knowledge Gaps and Future Perspectives 

### 8.1. Lack of Integrated Studies Combining All Three Bioactive Classes

A major limitation of the current literature is that lentil-derived peptides, resistant starch, and polyphenols are still investigated largely as separate functional entities rather than as interacting components within a shared food matrix. Although these fractions coexist in lentils and may contribute to overlapping, convergent, or potentially complementary effects in the gut, most available studies examine only one class at a time or evaluate whole lentils without fraction-level attribution. As a result, the field remains better positioned to infer matrix-level complementarity than to demonstrate true synergy in the strict experimental sense. This gap limits mechanistic interpretation at several levels, including microbial substrate use, short-chain fatty acid production, epithelial signaling, mucosal immune regulation, and redox balance.

Future research should therefore move beyond descriptive co-occurrence and toward formal interaction testing. In particular, the next generation of studies should be designed to determine whether combined lentil fractions produce effects that exceed co-directional additivity. This will require factorial or fraction-reconstitution designs in which peptides, resistant starch, and polyphenols are tested individually and in defined combinations within the same experimental platform. Such approaches should quantify interaction terms statistically, rather than inferring synergy from parallel beneficial outcomes alone. To be informative, these designs should be coupled with harmonized barrier-relevant endpoints, including tight junction-related markers, functional permeability measurements, inflammatory mediators, oxidative stress indicators, and microbiota-derived metabolites. Establishing whether lentil bioactives act additively, complementarily, or synergistically is therefore not simply a semantic refinement, but a necessary step toward a more rigorous understanding of lentil-associated gut health effects under physiologically relevant conditions.

### 8.2. Need for Multi-Omics, Human Trials, and Dose–Response Models

Another major knowledge gap lies in the limited mechanistic resolution of current evidence across digestion, fermentation, and host response. Existing studies suggest that lentil-derived fractions can influence microbial composition, fermentation products, epithelial inflammatory pathways, and barrier-associated readouts, but these processes are still rarely interrogated within an integrated systems framework. As a result, the links between food matrix transformation, microbial metabolism, and downstream host signaling remain incompletely defined. Future work should address this limitation through integrated multi-omics strategies embedded within interaction-focused experimental designs. Metagenomics, metatranscriptomics, metabolomics, and proteomics should be applied not as stand-alone descriptive tools, but as coordinated approaches to map how defined lentil fractions and their combinations reshape the microbiota–metabolite–host network. In particular, such studies should clarify whether combined exposure to peptides, resistant starch, and polyphenols produces distinct metabolic signatures, cross-feeding patterns, or host transcriptional responses that are not observed when each fraction is tested alone. The same logic applies to digestion and bioaccessibility: future studies should more clearly define the *in vivo* fate of lentil-derived peptides, phenolic metabolites, and fermentation-linked carbohydrate products, including their transformation, persistence, and site-specific activity across the gastrointestinal tract.

Equally important is the need for more rigorous human validation. At present, the evidence base remains weighted toward *in vitro* systems and animal models, with relatively limited human data and very few trials capable of distinguishing the effects of individual fractions from those of the whole food matrix. Future randomized controlled trials should therefore incorporate standardized lentil preparations, clearly defined intake levels, and clinically relevant endpoints related to intestinal permeability, inflammation, microbial metabolism, and symptom outcomes where appropriate. Dose–response modeling will also be essential. Rather than identifying only whether lentil intake is beneficial, future studies should determine whether combinations of lentil bioactives exhibit merely additive responses or whether supra-additive effects emerge across specific dose ranges. Such data will be critical for translating mechanistic findings into evidence-based dietary guidance, clinically relevant intervention strategies, and precision nutrition frameworks that account for interindividual differences in microbiome composition, dietary background, and metabolic responsiveness.

### 8.3. Future Potential for Lentil-Based Functional Foods and Nutraceuticals

The translational potential of lentils as a platform for functional foods and nutraceutical development is substantial, but future progress should be guided by evidence-supported formulation rather than presumed synergy. Processing strategies such as fermentation, enzymatic hydrolysis, fractionation, and controlled thermal treatment may help enrich specific bioactive fractions or improve their bioaccessibility, thereby creating more targeted lentil-based ingredients. However, the value of these approaches will depend on whether they can reproducibly enhance defined biological activities and whether such improvements translate into measurable barrier-relevant or metabolic outcomes *in vivo*. Future product development should therefore proceed along two parallel lines. The first is mechanistic standardization: processing methods should be optimized not only for compositional enrichment, but also for reproducible functional outputs linked to validated biomarkers. The second is clinical and nutritional relevance: candidate formulations should be evaluated within realistic dietary contexts, with attention being paid to palatability, stability, matrix effects, and target populations. This is particularly important if lentil-derived ingredients are to be positioned for applications such as gut health support, metabolic health formulations, healthy aging products, or medically oriented nutritional interventions. In these settings, claims should be anchored in demonstrated efficacy at defined doses and in well-characterized matrices, rather than extrapolated from isolated *in vitro* findings.

From a broader perspective, lentils remain highly attractive as a sustainable and nutrient-dense raw material in plant-based nutrition. Yet, the strongest path forward is not to assume that combined lentil bioactives are inherently synergistic, but to develop formulations in which such interactions are explicitly tested and, where present, quantitatively demonstrated. In this sense, the future of lentil-based functional foods lies in moving from compositional promise to mechanism-based validation, and from generalized health potential to precisely defined, evidence-supported applications.

## 9. Conclusions

Current evidence supports the concept that lentils function as a digestion-responsive bioactive matrix in which peptides, resistant starch, and polyphenol-rich fractions contribute to gut health through interconnected processes. During gastrointestinal digestion and colonic fermentation, lentil-derived protein-associated components, resistant starch, and phenolic compounds undergo sequential transformation, generating bioactive peptides, fermentable substrates, and microbiota-derived metabolites, particularly short-chain fatty acids. These linked processes influence microbial activity, metabolite production, and host epithelial responses in ways that are relevant to intestinal barrier function. Available studies indicate that lentil bioactives and their digestive products can modulate tight junction-related markers, reduce oxidative stress, and attenuate inflammatory signaling pathways, including NF-κB- and MAPK-associated responses, while phenolic-rich fractions in particular have also been linked to the activation of Keap1–Nrf2-mediated antioxidant defenses. Taken together, the current literature is more consistent with matrix-dependent complementary or convergent actions than with demonstrated synergy. At present, the clearest pathway-level evidence is available for phenolic-rich fractions, whereas fermentation-linked carbohydrate effects are more consistently supported by microbiota- and *in vivo*-associated outcomes, and protein- or peptide-related mechanisms remain comparatively underdefined. Important gaps remain, including the scarcity of integrated studies examining all three bioactive classes within the same platform, the lack of factorial designs capable of formally testing interaction effects, and the limited availability of robust human validation. Future research should therefore move beyond describing coexisting beneficial effects and toward determining, under physiologically relevant conditions, whether lentil bioactives act additively, complementarily, or truly synergistically.

## Figures and Tables

**Table 1:** Major lentil constituents associated with peptide precursors, resistant starch, and polyphenol-rich fractions.

Bioactive-Relevant Constituent	Representative Quantitative Value(s)	Main Localization/Distribution in Lentil Seed	Representative Subclasses/Fractions	Representative Processing-associated Change Relevant to Bioaccessibility	Reference
Protein/peptide precursor pool	Lentil seeds typically contain a protein range of 20–30%; seed storage protein represents up to 80% of total protein. Reported protein fractions vary across studies, with globulins in the range of 27–70%, albumins in the range of 4–61%, glutelins in the range of 3–47%, and prolamins in the range of 2–3%; the 7S/11S ratio is close to 3.	Seed storage proteins relevant to peptide generation are located mainly in the cotyledons	Mainly globulins and albumins; globulins include 7S vicilin/convicilin and 11S legumin.	Cooking time altered in vitro protein digestibility and proteolysis kinetics; in isolated cotyledon cells, the final readily bioaccessible protein fraction was in the range of ~30–37%, whereas in whole cooked lentils proteolysis generally increased with cooking time, although overly long cooking could reduce final protein digestibility in isolated cells.	[30,37,38]
Starch and resistant starch (RS) pool	In cooked lentils, total starch was in the range of 42.2–45.7% DW, apparent amylose: 7.9–12.4%, indirect-method RS: 8.0–16.7%, and direct-method RS: 2.5–11.9%. In isolated cotyledon cells, starch content was in the range of 60.8–62.6 g/100 g DM, compared with 44.7–46.9 g/100 g DM in whole cooked lentils.	Starch, and functionally the delivery of RS are primarily associated with the cotyledon cell matrix; direct hull-vs-cotyledon RS values were not reported	Total starch, apparent amylose, and RDS/SDS/RS fractions.	Whole lentils cooked for 15 min showed a lower starch digestion plateau (75.4 ± 5.4%) than isolated cotyledon cells at the same cooking time (101.4 ± 3.2%), indicating that matrix integrity and incomplete cell separation attenuated amylolysis. Longer cooking reduced physical barriers and increased starch digestibility.	[30,39]
Polyphenol-rich fraction	In green lentils, soluble total phenolics of dehulled/whole/hull fractions were 0.004/0.72/31.49 mg GAE/g, and insoluble-bound total phenolics were 0.004/0.39/53.88 mg GAE/g, respectively. In red lentil hulls, total phenolics reached 49.8 mg GAE/g DW, of which 55% were bound phenolics; condensed tannins were 15.83 mg CAE/g.	Polyphenols are strongly enriched in the hull/seed coat, relative to dehulled cotyledon-rich fractions.	Lentil polyphenols include phenolic acids, flavan-3-ols, flavonols, anthocyanidins, and proanthocyanidins/condensed tannins. Delphinidin and cyanidin were most abundant in hulls, whereas epicatechin and catechin were most concentrated in cotyledons.	Dehulling was the most effective treatment for reducing tannins, with reported reductions in the range of 89.46–92.99%, and it also decreased gallic acid, catechin, quercetin, and overall antioxidant activity.	[8,40,41,42]

Abbreviations: DW, dry weight; DM, dry matter; RDS, rapidly digestible starch; SDS, slowly digestible starch; RS, resistant starch; GAE, gallic acid equivalents; CAE, catechin equivalents.

**Table 2:** Effect of processing and digestion on lentil-derived bioactives (peptides, resistant starch, and polyphenols) and their gut-related functions.

Processing/Digestion Factor	Bioactive(s) Impacted	Key Change (↑/↓)	Experimental Model (What Was Tested)	Gut-Related Function/Outcome (Concise)	Reference
In vitro digestion of polyphenol-rich lentil hull extract	Polyphenols (digestion products)	Anti-inflammatory activity maintained after digestion	Simulated digestion + Caco-2 monolayer + Caco-2/RAW264.7 co-culture	↓ Inflammatory mediators; supports barrier integrity and anti-inflammatory signaling	[71]
Digestion products of Laird lentil hulls (free/bound phenolics)	Polyphenols	Bioactive digestive fraction (↑ anti-inflammatory)	HT-29 cells + digestive products characterized	↓ Inflammation via NF-κB inhibition and Keap1–Nrf2 activation (crosstalk)	[47]
Lentil hull polyphenols: release during digestion	Polyphenols	Release ↑ across digestion; antioxidant activity ↑	Simulated *in vitro* digestion + Caco-2 transport + rat bioavailability	↑ Antioxidant capacity in digesta; supports delivery of bioactives relevant to gut redox balance	[70]
Cooked red lentils in diet	RS + polyphenols (as colonic substrates)	Colonic microenvironment modulated dose-dependently	Mouse feeding (C57BL/6) with cooked red lentils	Microbiota composition/activity altered; ↑ barrier integrity/function	[49]
Red lentil pre-feeding before DSS colitis	Fiber/RS + phenolics (matrix)	Protective effect ↑	Mouse DSS colitis model with lentil supplementation	↓ Colitis severity; ↑ colon barrier function; ↓ inflammatory cytokines	[50]
Cooking time (short vs. longer)	Starch digestibility/RS-like behavior via intact structure	Short cooking → starch hydrolysis ↓	*In vitro* digestion of cooked lentils with different cooking times	Slower amylolysis, may ↑ starch delivery to colon (substrate for microbiota/SCFA)	[30]
High hydrostatic pressure (HPP) ± refrigerated storage	Resistant starch (RS)	RS content altered by HPP and storage	Legumes including green lentils	Non-thermal processing shifts RS → impacts fermentable carbohydrate availability in gut	[73]
High-pressure treatment of lentil starch dispersions	Resistant starch (RS)	RS ↑ (reported increase at high pressure)	Lentil starch under a range of 400–600 MPa	↑ Digestion resistance, suggesting ↑ colonic fermentation substrate	[59]
Extrusion-cooking severity (mild vs. severe)	Starch structure/digestibility	Gelatinization and functional changes (digestive functionality shifts)	Industrial extruded-cooked lentil flours	Processing alters digestibility kinetics, with possible implications for downstream substrate availability	[74]
Extruded corn–lentil formulations with added RS	Resistant starch + fiber/polyphenol-rich matrix	Higher-fortification formulations showed ↑ RS and ↑ fiber/phenolic-related components	Twin-screw extrusion of corn: lentil flour formulations followed by compositional/functional analysis	↑ Content of gut-relevant components (RS, fiber and phenolic-related compounds), implications for colonic fermentation or gut function were not directly tested, observed changes cannot be attributed to lentil alone	[75]
Gluten-free extruded snacks with lentil flour	Fiber/phenolics (matrix effects)	↑ Nutritional/functional profile improved (include digestion-relevant properties)	Product development and evaluation	Reformulation can ↑ gut-relevant components (fiber/phenolics), gut delivery or function was not directly assessed	[76]
Autoclaved lentil resistant starch during dynamic fermentation	Resistant starch (RS)	Microstructure evolution; microbiota metabolism shifts	Dynamic *in vitro* fermentation	Microbiota composition/metabolism altered; SCFA production patterns linked to RS structure	[27]
Fermented vs. unfermented lentil-containing flour prototypes (gel/bread) during static *in vitro* colonic fermentation	Protein–starch matrix/fermentable substrates	Food matrix effect > prior fermentation effect; SCFA production ↑	Static in vitro colonic fermentation using pooled fecal inoculum from older adults; microbiota profiling + SCFA analysis	Lentil-containing prototypes modulated microbiota composition and ↑ SCFA production	[77]
Fermentation of red lentil protein isolate (RLPI)	Peptides (hydrolysis products)	Proteolysis pattern depends on strains	Fermentation with LAB/yeasts; peptide/hydrolysis monitoring	Generated strain-dependent peptide pools with potential biological relevance after digestion	[53]
Fermented vs. raw red lentil protein isolate	Peptides	Protein digestibility ↑ after fermentation	*In vitro* digestibility + SHIME^®^ dynamic gut model	Gut microbiota responses assessed under peptide-rich protein fermentation conditions	[52]
Protein fermentation products (lentil vs. casein vs. wheat gluten)	Peptides + microbial metabolites	SCFA profile differs by protein source	TIM-2 *in vitro* colon + Caco-2/THP-1 assays	Lentil protein fermentation → SCFA ↑, cytotoxicity ↓, barrier damage ↓, IL-6 induction ↓	[78]
Co-fermentation (LAB + Bacillus subtilis natto)	Peptides/phenolics (functional components)	Functional and antioxidant components ↑	Co-fermented lentil product	↑ Antioxidant capacity (indicating potential gut-relevant compositional enhancement)	[79]
Lentil sprouts + probiotic (*L. plantarum* 299v) + digestion	Polyphenols	Gastric fraction phenolics ↑; GI digestion may reduce some	Lentil sprouts; buffer/gastric/GI fractions compared	Potentially bioaccessible phenolics may support gut redox/anti-inflammatory environment	[80]
Germination (24–48 h) of lentils	Phenolics + antioxidants; protein/starch digestibility	Phenolic/antioxidant bioaccessibility changes; digestibility shifts	Pulses including lentils; in vitro digestibility + bioaccessibility	Germination altered phenolic bioaccessibility and digestibility-related properties, indicating potential gut relevance	[81]
Enzymatic + microwave upcycling of lentil hulls	Polyphenols + oligosaccharides	Release ↑ (tech-dependent)	Lentil hull hydrolysates	↑ Fermentable oligosaccharides + phenolics, supports microbiota/SCFA potential	[40]
Fermentation + germination of lentil-based beverages	Polyphenols/antioxidant capacity	Antioxidant properties ↑ after bioprocessing	Lentil-based beverages; process comparison	↑Higher antioxidant activity relevant to gut oxidative stress context	[82]
Pre-gelatinization of red lentil flour (pasta)	Starch retrogradation/RS tendency	Retrogradation tendency ↑ vs. native	Pasta from pre-gelatinized red lentil flour	Pre-gelatinization modified starch retrogradation and pasta starch functionality, may ↑ colonic fermentable starch	[83]
Solid-state fermentation (SSF) and drying of lentil flour before gastrointestinal digestion	Peptides (digestion-derived peptide profile/ACE-inhibitory activity)	SSF altered peptide release; post-digestion ACE-inhibitory activity ↑ vs. unfermented flour, while drying partly restored activity	*In vitro* gastrointestinal digestion using standard and older-adult digestion models; peptide size/amino acid profile and ACE-inhibitory activity assessed	Processing modified peptide bioactivity; fermentation alone was not consistently beneficial	[84]
Lentil starch: amylose content variation	Resistant starch/digestibility	Digestibility properties vary with amylose	Lentil starch samples with different amylose	↑ Higher digestion resistance supports greater colonic fermentation substrate	[85]

Note: ↑ and ↓ indicate the direction of change relative to the study-specific control or comparator. ± indicates the presence or absence of refrigerated storage. SHIME^®^ = Simulator of the Human Intestinal Microbial Ecosystem. Because outcomes depend on processing conditions, matrix, and model system, the arrows summarize reported trends rather than universal effects.

**Table 3:** Representative lentil bioactives (peptides, resistant starch, and polyphenols), their mechanisms, and gut health outcomes.

Group	Bioactive Compound	Representative Lentil Bioactive	Experimental Model	Key Mechanism	Gut Health Outcome	Reference
Peptide	Protein/peptides (fermentation products)	Fermented lentil proteins → luminal extracts	TIM-2 in vitro colon + Caco-2 ± THP-1	↓ Cytotoxicity; ↓ barrier damage; lowest IL-6 induction; regulation linked to AhR signaling	↑ Barrier integrity; ↓ inflammatory signaling vs. animal protein comparators	[78]
Peptide	Peptides (fermented protein isolate)	Fermented red lentil protein isolate (FRLPI) peptides	SHIME^®^ dynamic gut model	Fermentation ↑ digestibility; bioactive peptide release; microbial shifts correlated with proteolysis	Gut microbiota shifts consistent with improved proteolysis and “gut health-linked” metabolites	[52]
Dietary fiber (Resistant starch)	Resistant starch	Lentil-derived RS	Humanized aging model in mice	Lentil RS remodeled the microbiome in a host- and sex-dependent manner; ↑ barrier-relevant outcomes	↑ Gut hyperpermeability/tight junction-related outcomes together with distinct microbiome shifts	[26]
Dietary fiber (Resistant starch)	Dietary fiber	Lentil hull soluble dietary fiber (SDF) (incl. cellulase-modified)	Colitis + behavioral deficit model in mice	As fermentable substrate → SCFAs; paper evaluates SDF impact in colitis/behavior outcomes	↓ Colitis indices; improvement in behavior deficits (gut–brain axis-type outcomes reported)	[100]
Dietary fiber (Resistant starch)	Resistant starch	Lentil-derived RS (autoclaved vs. untreated)	*In vitro* colonic fermentation (fecal cultures)	Different RS structures → different primary degraders; ALRS ↑ SCFAs; taxa shift (ULRS early ↑ *Bifidobacterium*; ALRS later ↑ *Ruminococcus*)	↑ SCFAs; microbiota remodeling dependent on RS structure	[27]
Dietary fiber (Resistant starch)	Fiber/oligosaccharides	Lentil hull hydrolysate enriched in oligosaccharides + free phenolics	Ingredient generation (upcycling) + gut fermentation relevance	↑ Increases fermentable oligosaccharide pools and free phenolics (prebiotic-relevant substrate)	Supports potential prebiotic substrate availability for gut microbes	[40]
Polyphenols	Polyphenols	Red/green lentil hull extracts (RLE/GLE) + digestion product (RLD)	Caco-2 monolayer + Caco-2/RAW264.7 co-culture (LPS)	↓ iNOS/COX-2; ↓ NO/IL-6/IL-1β; inhibits MAPK + NF-κB; digestion products can be absorbed and support barrier	↑ Barrier maintenance; ↓ inflammatory mediators	[71]
Polyphenols	Polyphenols	Lentil hull polyphenols (bioaccessible fraction)	In vitro digestion + Caco-2 transport	Demonstrates bioaccessibility + transepithelial transport/bioavailability of hull polyphenols	Supports feasibility of gut exposure + uptake (bioavailability evidence)	[70]
Polyphenols	Polyphenols	Free/bound phenolics of Laird lentil hulls + digestive products	HT-29 cells (inflammation model)	Digestive products act via crosstalk between NF-κB and Keap1-Nrf2 signaling	↓ Inflammatory signaling; antioxidant defense modulation	[47]
Polyphenols	Polyphenols	Phenolics from cooked green lentil (released during GI digestion)	Simulated UGI digestion + cell-based assays	Soluble phenolics ↑ across gastric→intestinal phases; measurable bioaccessibility	↑ Antioxidant/↓ inflammatory readouts in cell assays	[119]
Polyphenols	Polyphenols (waste streams)	Lentil hull/waste extracts (e.g., BEVa)	LPS-activated Caco-2 + intestinal contractility assays	↓ inflammatory response in Caco-2; spasmolytic effects (gut function)	Supports intestinal health maintenance; potential barrier dysfunction support	[101]
Polyphenols	Polyphenols/antimicrobial	Cooked lentil extract vs. enterotoxigenic E. coli	Epithelial infection model (ETEC)	Lentil extract reduces pathogen virulence/host inflammatory damage	↓ pathogen-induced inflammatory effects; supports epithelial protection	[120]
Polyphenols	Saponins	Red lentil saponin-rich extract → soyasapogenol B	In vitro colonic fermentation (human fecal microbiota)	Microbiota transforms saponins → sapogenins; modulates bacterial groups; antimicrobial effect noted	Microbiota modulation + sapogenin generation (gut bioavailability/biotransformation)	[121]
Combination	Whole food (legume matrix)	Daily lentil consumption	Human intervention (12-week)	Human metabolic improvements; gut-linked mechanisms discussed alongside fiber/RS fermentation potential	Health improvements reported with sustained lentil intake	[103]
Combination	Whole food (polyphenols + fiber + RS)	Cooked red lentils (dietary intervention)	Healthy male mice	↑ SCFA-producing taxa; ↑ fecal SCFAs; ↑ SCFA receptors (GPR41/43); ↑ tight/adherens junction mRNAs (ZO-1, etc.)	↑ Barrier integrity/function; ↑ microbial diversity; ↑ SCFAs	[49]
Combination	Whole food (polyphenols + fiber + RS)	Red lentil supplementation (pre-feeding)	DSS colitis mouse model	Lentil pre-feeding attenuates inflammation; supports barrier function (colitis context)	↓ Clinical symptoms; ↑ barrier function; ↓ colonic inflammatory cytokines	[50]
Combination	Fiber + polyphenols (gut–liver axis)	Green lentil hulls (GLH) supplement	NAFLD model (gut–liver axis framing)	Proposed integrated effect of fiber + polyphenols on intestinal and hepatic metabolism	Improved gut health indicators linked to ↓ NAFLD risk (gut–liver axis)	[102]
Combination	Fermented lentil beverage matrix	Lentil-based fermented beverages	Multiple probiotic strains (product + microbiology)	Fermentation changes bioactives (acidification/biotransformation) relevant to gut exposure	Improved functional profile consistent with gut-targeted foods	[122]

Note: ↑ and ↓ indicate the direction of change relative to the study-specific control or comparator; RLD, digestive products of red lentil hulls; ETEC, enterotoxigenic Escherichia coli; BEVa, microwave-assisted ethyl acetate extract of Eston green lentil hulls; NAFLD, non-alcoholic fatty liver disease; DSS, dextran sodium sulfate.

**Table 4:** Structured summary of in vitro, animal, and human studies relevant to lentil bioactives and gut barrier mechanisms.

Lentil Bioactive/Matrix	Model/Design	n	Intervention	Primary Endpoint(s)	Barrier Endpoint(s)	Microbiome Endpoint(s)	Main Finding(s)	Limitation(s)	Reference
Lentil hull phenolic digestive products	*In vitro* digestion; RAW264.7, Caco-2 monolayer, Caco-2/RAW264.7 co-culture	NR	Simulated digestion; RLD under LPS challenge	NO, IL-6, IL-1β, iNOS, COX-2, MAPK/NF-κB	Papp; claudin-1; ZO-1	None	Anti-inflammatory effect with improved epithelial barrier-related readouts	*In vitro* only; exposure levels may not reflect in vivo availability	[71]
Free/bound lentil hull phenolic digestive products	*In vitro*; TNF-α-stimulated HT-29 intestinal epithelial model	NR	25–100 μg/mL digestive products	IL-6, IL-8, IL-1β, COX-2, NF-κB, Keap1–Nrf2, HO-1, NQO-1	Indirect barrier relevance only	None	Pathway-level anti-inflammatory effect via NF-κB suppression and Keap1–Nrf2 activation	Cancer cell line; no direct permeability endpoint	[47]
Lentil extract (fiber-containing product)	*In vitro*; ETEC-infected Caco-2/HT29-MTX co-culture; complementary fecal microbiota model	NR	2 g/L fiber equivalent; 3 h pre-treatment; MOI 100	Toxin production, IL-8, adhesion, mucus-related genes, virulence genes	TEER; Transwell permeability	No significant effect on ETEC colonization in complex fecal background	Lentil extract reduced heat-labile toxin (LT) production and IL-8; strengthened barrier function in the mucus-secreting intestinal model	Infection-specific model; not a general barrier-restoration study	[120]
Lentil protein fermentation products (luminal extracts)	TIM-2 with pooled healthy adult microbiota; downstream Caco-2 and Caco-2/THP-1 assays	Duplicate TIM-2 runs	10 g/day protein-supplemented SIEM for 72 h	SCFAs, BCFAs, cytotoxicity, IL-6	Less barrier damage reported in downstream epithelial assay	Fermentation metabolite profile	Lentil protein fermentation products were less detrimental than casein/VWG comparators	Excess-protein design may not reflect typical lentil intake	[78]
Lentil hull waste extracts; BEVa most active	*In vitro* LPS-activated Caco-2 cells + ex vivo guinea pig ileum and proximal colon	NR	Extract pre-treatment 1 h, then LPS 1 μg/mL for 24 h; ex vivo 0.1–10 mg/mL	TLR4, iNOS, IL-1, IL-10; longitudinal/circular tone, transit speed, pain, mixing	Indirect barrier-supportive only	None	BEVa downregulated TLR4/iNOS/IL-1, upregulated IL-10, and showed spasmolytic activity in ileum/colon	No direct TEER/TJ/permeability endpoint; ex vivo motility data are not equivalent to in vivo barrier protection	[101]
Lentil acetonitrile–water extract	*In vitro*; LPS-induced Caco-2 epithelial injury model	≥3	Lentil extract 2.5 mg/mL + LPS 10 μg/mL	TNF-α, NF-κB, IL-1β, IL-8, TRPA1, TRPV1, AQP8, TLR4	TEER; occludin; ZO-1	None	Preserved epithelial integrity and reduced inflammatory signaling	Mono-culture model; no immune or microbiome component	[133]
Whole cooked red lentils	*In vivo*; healthy feeding study; 5-week-old male C57BL/6 mice	12/group	5%, 10%, or 20% lentil powder for 3 weeks; 0.7% pectin comparator	Colonic microenvironment; fecal SCFAs; gene expression	ZO-1; claudin-2; E-cadherin; GPR41/43	16S fecal microbiota; α-diversity; SCFA-producing taxa	Dose-dependent improvement in colonic microenvironment; 20% strongest	Healthy model only; male only; whole food effects not attributable to one fraction	[49]
Whole red lentils	*In vivo*; acute DSS colitis; 5-week-old male C57BL/6 mice	12/group/time point	20% lentil diet for 3 weeks pre-feeding; then 2% DSS for 5 days	DAI, histology, cytokines, serum LBP	Occludin; IL-22; Relmβ; serum LBP	Pre-DSS cecal SCFAs	Pre-feeding attenuated colitis severity and improved repair-/barrier-related markers	Acute chemically induced model; male only	[50]
Modified lentil hull soluble dietary fiber	*In vivo*; DSS colitis + gut–brain axis model; male C57BL/6J mice, 6–8 weeks	12/group	LSDF 500 mg/kg/day or HSDF 1000 mg/kg/day; 7 d pre-DSS + 7 d 2% DSS	Behavioral tests, colon/brain histology, inflammatory markers, metabolomics	Indirect only	16S rRNA; harmful vs. beneficial taxa shift	SDF improved colitis and behavioral deficits; LSDF outperformed HSDF	No direct permeability/TJ endpoint	[100]
Lentil-resistant starch	*In vivo*; humanized aging model; 60-week-old C57BL/6J mice with pooled older human microbiota; both sexes	14–16/group	5% *w*/*w* lentil RS in western-style HFD for 20 weeks	Intestinal health and inflammatory outcomes	Gut hyperpermeability; claudin-1; claudin-4	Sex-dependent microbiome shifts	Lentil RS supported microbiota-linked barrier preservation; LEN particularly strong for leakiness/TJ markers	Purified RS, not whole lentil; sex-dependent responses	[26]
Whole green lentils	RCT; adults 18–70 y with increased waist circumference and elevated postprandial triglycerides	38 completers	0 vs. 980 g/week whole green lentils for 12 weeks	Fasting/postprandial lipids, glucose, inflammatory markers, GI symptoms	Not directly assessed	Not primary	Improved fasting cholesterol and postprandial glucose/inflammatory responses without GI distress	No direct barrier endpoint	[103]
Whole green lentils	Single-blind parallel RCT; adults 18–70 y, metabolically at risk, non-diabetic, increased waist circumference	30 completers	0, 300, or 600 g/week cooked green lentils for 8 weeks	Glucose/insulin responses, HOMA-IR, satiety, GI symptoms	Not directly assessed	Not primary	Reduced progression of insulin resistance with low GI symptom burden	No direct barrier endpoint	[137]
Whole green lentils	Parallel RCT; metabolically at-risk adults with elevated waist circumference and triglycerides	36	0 vs. 980 g/week whole green lentils for 12 weeks	16S rRNA composition; postprandial inflammation, lipids, glycemia	Not directly assessed	Family-, genus-, and OTU-level shifts; increased Bifidobacterium	Long-term lentil intake shifted gut microbial composition and related metabolic responses	No direct permeability or tight junction endpoint	[138]

Table note: TEER, transepithelial electrical resistance; TJ, tight junction; Papp, apparent permeability coefficient; SCFAs, short-chain fatty acids; BCFAs, branched-chain fatty acids; DSS, dextran sodium sulfate; LPS, lipopolysaccharide; LBP, lipopolysaccharide-binding protein; RLDs, digestive products of red lentil hulls; ETEC, enterotoxigenic Escherichia coli; BEVa, microwave-assisted ethyl acetate extract of Eston green lentil hulls; DAI, disease activity index; LSDF, low-dose soluble dietary fiber; HSDF, high-dose soluble dietary fiber; HFD, high-fat diet; HOMA-IR, homeostatic model assessment of insulin resistance; OTU, operational taxonomic unit; NR, not clearly reported or not retained in the compressed format.

## Data Availability

No new data were created or analyzed in this study.
